# Mobile genetic elements drive a plasmid fusion and deletion lifecycle shaping evolution and antimicrobial resistance

**DOI:** 10.1038/s41467-026-77678-8

**Published:** 2026-09-15

**Authors:** Thomas Ipoutcha, Yiqing Wang, Eduardo P. C. Rocha, José R. Penadés

**Affiliations:** 1 Department of Infectious Disease, Imperial College London, London, UK; 2 Centre for Bacterial Resistance Biology, Imperial College London, London, UK; 3 Institut Pasteur, Université de Paris Cité, CNRS UMR3525, Microbial Evolutionary Genomics, Paris, France; 4 Veterinary School, Universidad CEU Cardenal Herrera, Alfara del Patriarca, Spain

**Keywords:** Bacterial evolution, Molecular evolution

## Abstract

Plasmids are key drivers of bacterial adaptation, yet the mechanisms that generate their diversity remain poorly understood. Here, we show that mobile genetic elements (MGEs) orchestrate a fusion-deletion life cycle that repeatedly remodels plasmids in *Staphylococcus aureus*. Large-scale genomic analyses reveal that multireplicon plasmids are widespread and strongly enriched in transposases. Using experimental assays, we demonstrate that rare MGE-mediated fusion events, via homologous recombination or transposition, combine distinct plasmids into single multireplicon elements, expanding gene content and transfer potential. Antibiotic pressure selectively enriches these fused plasmids, rescuing bacterial populations under stress, whereas opposing selective forces, including phage predation, favour deletion derivatives that preserve essential functions and phage transmissibility. This cyclical process generates dynamic plasmid repertoires with conserved backbones and diverse accessory modules. We propose that MGE-driven fusion-deletion cycles represent a general principle of plasmid evolution, explaining the rapid emergence and persistence of multidrug-resistant plasmids across bacterial pathogens.

## Abstract (web summary)

Plasmids drive bacterial adaptation, but how their diversity arises has remained unclear. Here, they show that in *Staphylococcus aureus*, mobile genetic elements repeatedly fuse and delete plasmids, reshaping gene content under competing pressures and accelerating the emergence and persistence of multidrug resistance.

## Introduction

Plasmids are central drivers of bacterial evolution, mediating the horizontal transfer of genes that confer antibiotic resistance, virulence and metabolic versatility^1,2^. Their mobility enables rapid adaptation across ecological and clinical contexts and has been a major force in the emergence and global spread of multidrug-resistant pathogens, particularly among the ESKAPE bacteria^3^. As antimicrobial resistance (AMR) continues to escalate worldwide^4,5^, identifying the evolutionary principles that govern plasmid diversity has become a critical priority.

Across bacteria, plasmids adopt distinct mobility strategies, ranging from large self-transmissible elements (conjugative) to smaller mobilizable plasmids that rely on other mobile elements for dissemination^6^. Conjugative plasmids encode the full machinery required for self-transfer, whereas mobilizable plasmids carry only partial transfer functions, such as an origin of transfer and sometimes a relaxase, and depend on a conjugative plasmid to move by conjugation^7^. Some plasmids, known as non-mobilizable (also referred to as non-transmissible) plasmids, lack any known transfer mechanism and depend on phage-mediated transduction for their transfer. Conjugative plasmids are generally large (> 25 kb in Firmicutes)^8^, reflecting the need to encode the genes for the mating pair formation system. In contrast, across all analysed species, mobilizable and non-transmissible plasmids display a bimodal size distribution^1^, with peaks corresponding approximately to the size of temperate phage genomes (~ 40–45 kb) and phage satellites (~ 10 kb) in *Staphylococcus*^9,10^. This bimodality is consistent with the idea that smaller plasmids lacking conjugation-related genetic elements can nevertheless be mobilised via phage- or satellite-mediated generalized transduction^11^.

These mechanisms of transfer impose strong physical and ecological constraints on plasmid size, creating a fundamental trade-off between mobility and gene content^1^. Smaller plasmids can be efficiently transferred by phages but encode limited accessory functions, whereas larger plasmids carry extensive adaptive cargo but rely almost exclusively on conjugation. Despite these constraints, natural plasmid populations display remarkable diversity in size, structure and gene content, raising a central unresolved question: how is plasmid diversity generated and maintained in the face of strong selective and physical limitations? Although plasmids can gain, lose and rearrange genetic material, the evolutionary principles governing these processes remain poorly understood^12^.

Multireplicon plasmids have been described for decades and result from plasmid fusion events. Such plasmids have been observed in several pathogenic genera, including *Klebsiella*^13–17^, *Acinetobacter*^18^, *Salmonella*^19^, *Citrobacter*^20^, *Enterococcus*^21^ and *Staphylococcus*^22^. Historically, multireplicon plasmids were largely regarded as rare or exceptional outcomes of plasmid evolution, documented mainly as isolated case studies rather than as a pervasive evolutionary strategy.

Recent comprehensive bioinformatic analyses have revealed that multireplicon plasmids are widespread and play a major role in the emergence of clinically important plasmids^23,24^. These studies have shown that plasmids have progressively accumulated AMR genes^24–26^, and that multireplicon plasmids have become especially prevalent since the introduction and widespread use of antibiotics^24^. However, despite growing recognition of their importance, the prevalence, evolutionary significance and mechanistic basis of plasmid fusion remain poorly resolved in many bacterial species, including *Staphylococcus aureus*, and it is unclear how rare fusion events become evolutionarily significant, how they are integrated over time, or whether multireplicon plasmids represent stable endpoints or transient intermediates in plasmid evolution.

Here, we propose that plasmids follow a population-level evolutionary cycle driven by mobile genetic elements. In this life cycle, rare fusion events transiently expand plasmid size, gene content and mobility, whereas opposing selective forces, including bacteriophages, promote deletion and resolution, eliminating excess DNA while preserving essential functions and transmissibility. Unlike the life cycles of bacteriophages or other mobile elements, which largely preserve element identity across generations, plasmid evolution results in rapid diversification. Fusion generates genetic novelty, and subsequent deletion or resolution produces multiple distinct descendants rather than a single reverting form. This recurrent remodelling, rather than preservation of a core set of genes, fosters the exploration of genetic diversity within plasmid populations while maintaining essential genes for plasmid replication. Our results therefore provide a mechanistic explanation for how plasmids achieve extensive genetic diversity despite strong constraints on size and mobility, reconciling rapid adaptation with long-term evolutionary stability.

## Results

### Plasmid diversity and fusion signatures in *S. aureus*

To determine how plasmid diversity is generated and maintained despite strong constraints on size and mobility, we analysed plasmids from *S. aureus*, a species in which plasmid-mediated gene transfer is central to adaptation, virulence and AMR^2^. The coexistence of conjugative, mobilizable and non-transmissible plasmids within the same populations provides a natural system to assess how different mobility strategies, genetic architectures and fusion signatures are distributed at scale.

**Fig. 1:**
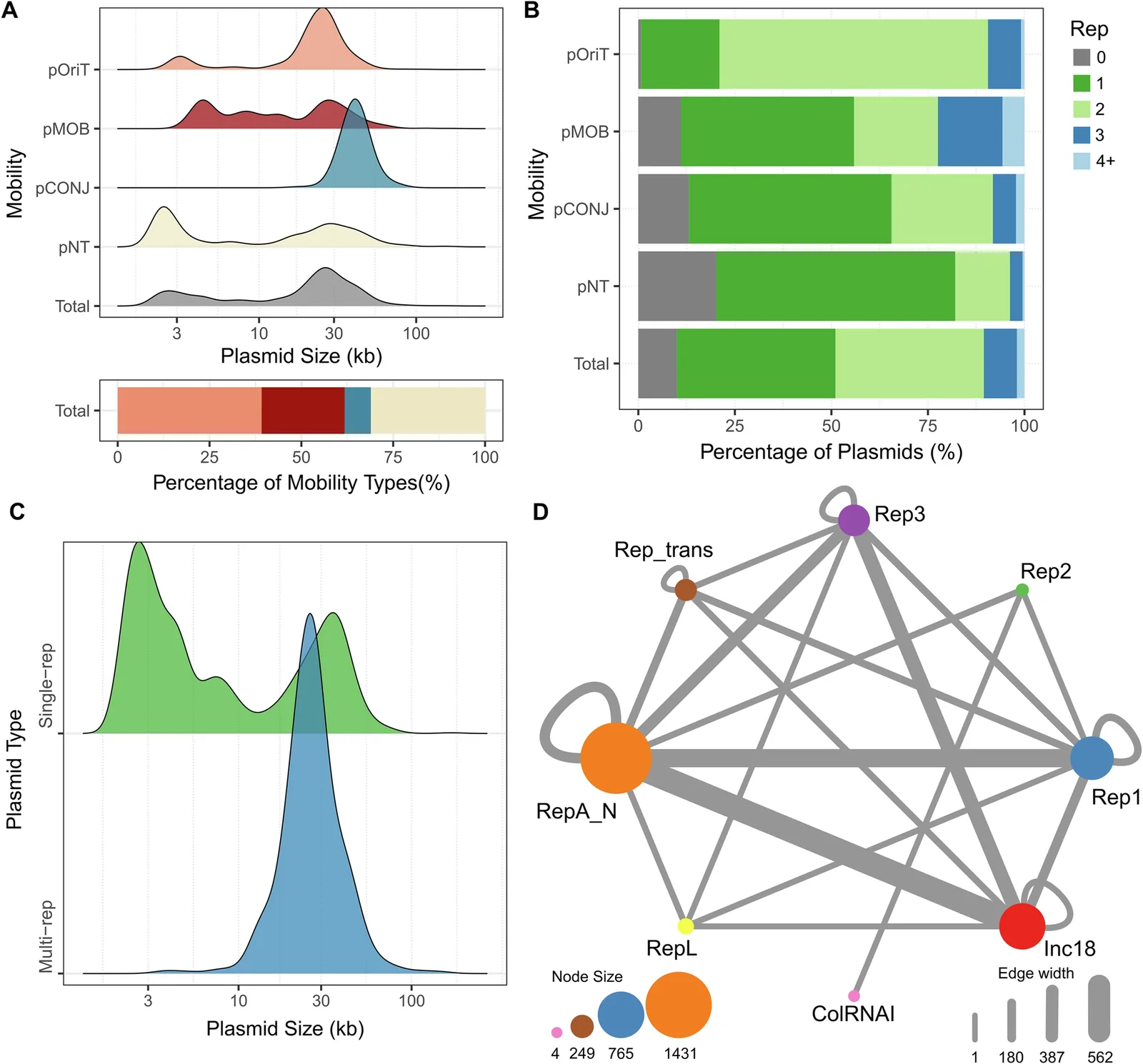
Mobility classes and replicon architectures of *Staphylococcus* plasmids. Plasmids were classified into four mobility types: pCONJ (conjugative), pMOB (mobilizable), pOriT (carrying *oriT* sequences) and pNT (non-transmissible). **A** Density distribution of plasmid sizes (log10 transformed) across mobility classes (top) and relative proportion of plasmids in each mobility category (bottom). Approximately 69% of plasmids encode genetic determinants associated with conjugation-related mobility. **B** Distribution of replicon number across plasmid mobility classes. Plasmids carrying more than four replicons were grouped as “4+”, whereas plasmids lacking identifiable replicons were assigned “0”. Multireplicon plasmids are enriched among pOriT plasmids (chi-square test, *P* < 0.001; pairwise Fisher’s exact test, odds ratio > 7, Benjamini–Hochberg adjusted *P* < 0.001). **C** Size distribution of single- and multireplicon plasmids. Single-replicon plasmids display a bimodal size distribution, whereas multireplicon plasmids exhibit a predominantly unimodal distribution. **D** Replicon co-occurrence network for multireplicon plasmids. Nodes represent replicon families, with node size proportional to family frequency. Edges indicate co-occurrence within the same plasmid, and edge thickness reflects the number of co-occurrence events. RepA_N, Rep3 and Inc18 replicons are frequently associated with multireplicon architectures. Rolling-circle replicons: Rep1, Rep2, RepL and Rep_trans. Theta-replicating plasmids: RepA_N, Inc18, Rep3 and ColRNAI.

We compiled a dataset of 2564 complete *Staphylococcus* plasmids from the PLSDB database, including 1649 from *S. aureus*. Plasmids were classified according to predicted mobility and replicon content (Fig. 1). Among plasmids carrying at least one identifiable Rep gene (2308/2564), 7% were conjugative (159), 22% mobilizable (516), 43% encoded an origin of transfer (997) and 31% were non-transmissible (636) (Fig. 1A).

Unexpectedly, nearly half of all plasmids (49%, 1255/2564) carried more than one identifiable replicon sequence. We refer to these sequence-defined elements as multireplicon plasmids; this classification does not imply that all detected replicons are intact or functionally active. Additional incomplete or inactive replicons may instead represent signatures of historical plasmid fusion events (Fig. 1B). Fusion signatures were particularly enriched among plasmids that rely on conjugation for transfer (chi-square test, *p*-value < 0.001), particularly among plasmids containing just an origin of transfer for conjugation (pOriT) (Fisher’s exact test, odds ratio > 7, *p*-value < 0.001, B–H adjusted) whereas they were substantially less frequent among non-transmissible plasmids (Fig. 1B). Consistent with plasmid co-integration, multireplicon plasmids were significantly larger than single-replicon plasmids (Fig. 1C), indicating that fusion contributes directly to plasmid expansion.

Analysis of replicon families revealed that the presence of multiple replicons within a single plasmid, considered a hallmark of plasmid fusion, is widespread and encompasses nearly all possible combinations of replicons (Figs. 1D and S1A). Small plasmid families, including RepL and Rep_trans, were typically restricted to 2–8 kb when present as a single replicon, whereas larger plasmids carrying these replicons were almost exclusively multireplicons that had incorporated them (Supplementary Data 1 and Fig. S1C, D). Plasmids encoding RepA_N, Rep3 and Inc18 families showed significantly higher frequencies of fusion (Fisher’s exact test, *p*-value < 0.001), suggesting that certain replicon types play a central role in plasmid co-integration and diversification. Interestingly, plasmids with such replicons tend to be larger and mobilizable by conjugation (Fig. S1B). Together, these results show that plasmid fusion is frequent in *S. aureus*, providing a potential mechanism for generating genetic diversity.

### Association of mobile genetic elements with plasmid fusion

Plasmid fusion is favoured by the presence of identical or highly similar sequences in distinct plasmids. Because insertion sequences (IS) are involved in plasmid evolution and are overrepresented in plasmids of clinical importance^27,28^, transposases are strong candidates for mediating such co-integration events. Indeed, IS elements, particularly those of the IS*6* family (e.g., IS*257*), were shown to generate transient plasmid co-integrates in *Escherichia coli*^29–33^ and *S. aureus*^22,34,35^. To determine whether IS-mediated fusion is a widespread feature of plasmid populations, we analysed the *Staphylococcus* plasmid dataset for the presence of ISs.

BLASTn analysis revealed that 40% of *Staphylococcus* plasmids carry IS*6*, with a significant enrichment among multireplicon plasmids (Fisher’s exact test, *p*-value < 0.001. 58% in plasmids with two replicons and up to 98% in plasmids with four replicons, compared with 22% in single-replicon plasmids) (Fig. S2B). Conjugative and *oriT*-encoding plasmids, both of which display elevated fusion frequencies, were also enriched for IS*6* (52 and 50%, respectively) (Fig. S2C). In contrast, RepL plasmids showed low frequencies of both IS*6* carriage (12%) and fusion signatures (10%) (Figs. S1A and S2A). Conversely, replicon families with high fusion prevalence, such as RepA_N, were strongly enriched for IS*6* (67%).

The Mu-family transposase encoded in IS*Bli29* (hereafter IS*Bli29* ORF4 transposase referred as IS*Bli29*), was one of the most abundant IS in *Staphylococcus* plasmids and showed a similar association with plasmid fusion. IS*Bli29* was rare in single-replicon plasmids (3%) but enriched in multireplicon plasmids (18–26%) (Fig. S2B). IS*Bli29* was particularly prevalent in Rep3 plasmids (39%), a family in which IS*6* elements are less common (27%) (Fig. S2A), yet fusion frequencies are high (77%) (Fig. S1A). Both IS*6* and IS*Bli29* were associated with increased plasmid size (Fig. S2D), consistent with their role in promoting plasmid co-integration^28,36^.

We then assessed the impact of plasmid mobility, size and presence of ISs on the probability that the plasmid is multireplicon using a generalized linear model and found that all variables contributed significantly and positively to the latter, i.e., multireplicon plasmids tend to have more ISs, larger size and are more likely to be mobile or mobilizable by conjugation (Table 1). Together, these results identify transposable elements as major contributors to plasmid fusion, providing a mechanistic link between the widespread occurrence of multireplicon plasmids and the enrichment of ISs in plasmid genomes.

**Table 1:** Binomial generalized linear model for multi-/single-replicon plasmids

Predictor	Estimate	95% CI	*z*-value	*P*-value
Intercept	−14.67	[−16.42, −12.99]	−16.81	<0.001
Log10(Size)	2.98	[2.59, 3.39]	14.61	<0.001
IS_presence	1.36	[1.11, 1.62]	10.41	<0.001
Mobility	2.06	[1.79, 2.34]	14.63	<0.001

Plasmids encoding multiple-/single-replicon were analyzed using a binomial generalized linear model (glm) with a logit link. *P*-values were obtained from a multivariable logistic regression using two-sided Wald tests. Plasmid size shows a significant effect on the probability of a plasmid being multireplicon, IS_presence (presence of either IS*6* or IS*Bli29*) also had an independent significant effect. Compared to plasmids lacking mobility (pNT), plasmids with conjugation-related mobility (pOriT, pMOB and pCONJ) show an independent significant effect. *P*-values: Intercept = 2.13889e-63, Log10(Size) = 2.23685e-25, IS_presence = 2.30521e-48, Mobility = 1.79359e-48.

### Plasmid families with conserved backbones show evidence of modular evolution

**Fig. 2:**
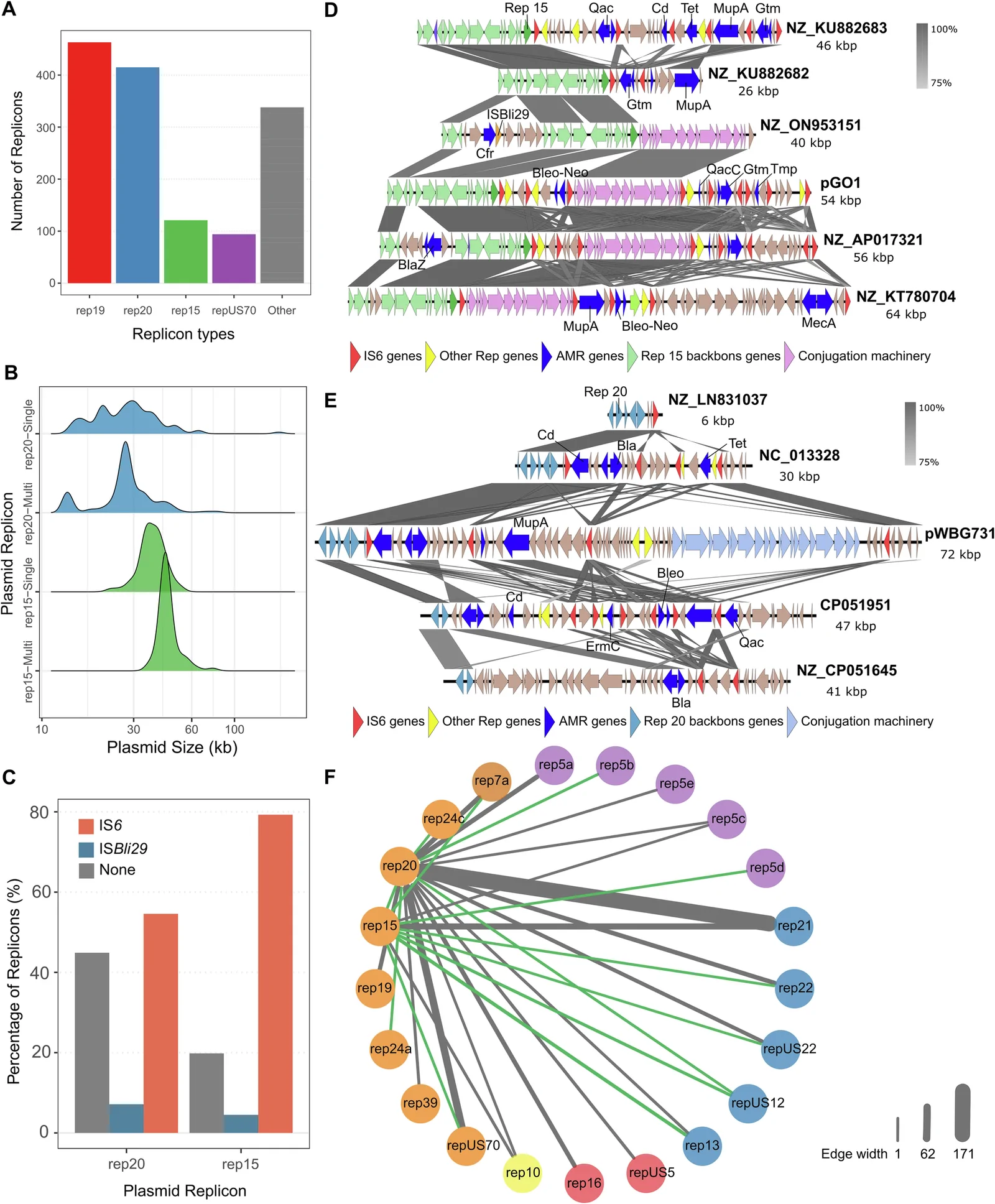
Conserved backbone structure and modular diversification of rep15 and rep20 plasmids. **A** Distribution of replicon types within the RepA_N family. Rare replicon types were grouped as “Other”. **B** Size distribution of plasmids encoding rep15 or rep20 replicons. **C** Prevalence of IS6 and ISBli29 insertion sequences in rep15- and rep20-containing plasmids. **D**, **E** Representative sequence alignments of rep15 (**D**) and rep20 (**E**) plasmids. Plasmids were selected to capture variation in size, antimicrobial resistance (AMR) gene content, conjugation machinery and additional replicons. Conserved backbone regions coexist with variable accessory modules. Replication genes are shown in yellow, IS6 elements in red and AMR genes in blue. Resistance annotations: Gtm gentamicin, Cd cadmium, Tmp trimethoprim, Erm erythromycin, Bleo bleomycin, MupA mupirocin, Bla β-lactam, Qac quaternary ammonium compound resistance. **F** Replicon co-occurrence network for rep15- and rep20-containing plasmids. Nodes represent replicon types coloured by family. Edges indicate co-occurrence within the same plasmid, and edge thickness reflects the number of co-occurrence events. Green edges indicate plasmid combinations with median size >45 kb, whereas grey edges indicate combinations with median size ≤45 kb.

To investigate how plasmids diversify while maintaining conserved core functions, we asked whether plasmids sharing the same replicon type (the same replication machinery) also vary in size and gene content. If so, this would indicate that plasmids undergo repeated gain and loss of accessory genes over time. We focused on plasmids carrying the RepA_N replicon, which display a high prevalence of multireplicon architectures (Fig. S1A). Within this group, we analysed two representative subfamilies, Rep15 and Rep20 (Fig. 2A), both of which frequently carry IS*6* (Fig. 2B) and exhibit substantial size variability (Fig. 2C).

Comparative sequence analyses revealed highly conserved backbones around the rep gene regions within each subfamily. In contrast, we found important variation in accessory genes, particularly AMR genes in the plasmids (Fig. 2D, E). Many accessory modules consisted of complete or partial segments from other plasmids with diverse replicon types (Fig. 2D–F). These modules were commonly flanked by IS*6*, and often included additional ISs, implicating transposases in both their acquisition and subsequent rearrangement. In the Rep15 subfamily, 90% of plasmids retained a conjugation module, whereas a minority lacked this region, consistent with deletion events (Supplementary Data 1 and Fig. 2D). Conversely, Rep20 plasmids were predominantly *oriT*-encoding or mobilizable, but a small subset carried conjugation machinery, consistent with sporadic acquisition events (Supplementary Data 1 and Fig. 2E).

Despite frequent fusion and rearrangement, plasmid sizes in both Rep15 and Rep20 remained restricted, with very few exceeding 45 kb (Fig. 2B). This pattern extended to the full *Staphylococcus* plasmid dataset, where only 6% of plasmids (162/2564) were larger than 45 kb (Supplementary Data 1).

Together, these observations indicate that plasmids with conserved replication backbones undergo continuous, modular remodelling through the gain and loss of accessory regions. This process can generate important diversity in AMR gene combinations while preserving core plasmid functions. The mechanisms underlying this modular evolution are explored in the following sections.

### Experimental evidence for plasmid fusion

To directly test the mechanisms driving plasmid fusion, we selected naturally occurring plasmids that carry at least one IS*257* (IS6 family) element and belong to replicon families showing strong bioinformatic signatures of fusion (Figs. S1A and S2A). pGO1 is a conjugative plasmid from the Rep15 family^37^ for which smaller conjugative (p.EvoF) and non-conjugative (p.Evo) derivatives have been described in ref. ^11^. pWBG731 (p.731) is a conjugative plasmid carrying two functional replication genes, Rep20 and Rep24A^22^, along with a smaller non-conjugative derivative (p.144) retaining only Rep24A. As a representative small, non-transmissible plasmid, we used pUR3912 (p.3912), which belongs to the Rep13 family^38^ (Fig. S3A).

To determine whether plasmid fusion can occur at the population level, we introduced pairs of large conjugative and small non-transmissible plasmids into the same *S. aureus* strain (pGO1 or p.731 together with p.3912), allowed cultures to propagate and extracted plasmid DNA. We designed PCR primers to specifically detect fusion junctions generated by homologous recombination between IS257 elements (Supplementary Data 5). Using this approach, we amplified the expected fusion products and confirmed their junction sequences by Sanger sequencing (Fig. S3B).

These results demonstrate that plasmid fusion can occur naturally through homologous recombination at IS*257* sites. However, while PCR detection establishes the occurrence of fusion events, it does not inform on their frequency within the population or the selective forces that promote their persistence, questions that we address in the following experiments.

### Antibiotic selection enriches fused plasmids

Plasmid fusion can occur naturally, but such events are intrinsically rare, in part because the homologous recombination rate is low in *S. aureus*^39^. This rarity contrasts sharply with the high prevalence of multireplicon plasmids observed in genomic databases, suggesting that fused plasmids must be strongly selected in nature to reach detectable frequencies.

**Fig. 3:**
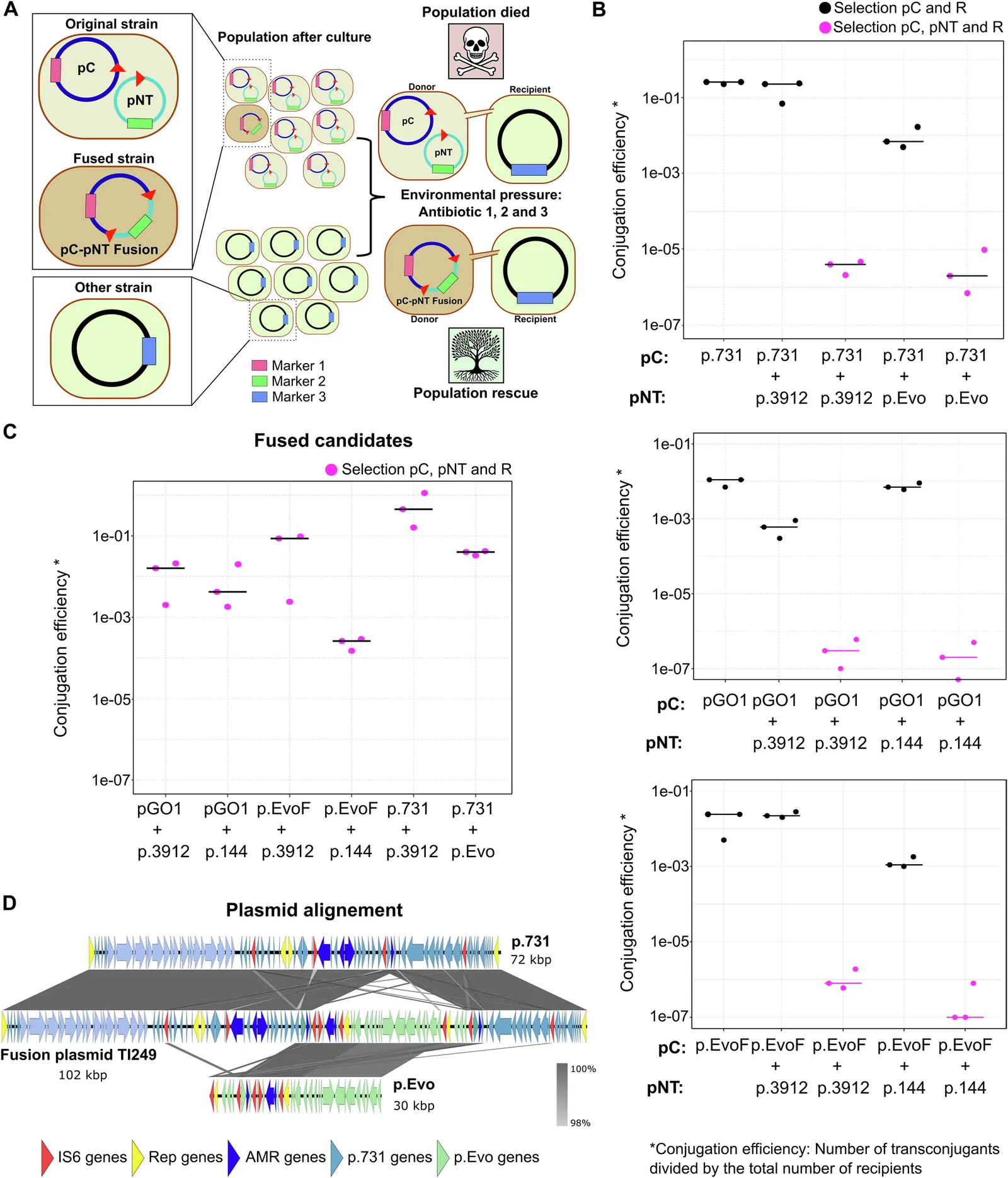
Antibiotic selection enriches rare plasmid fusion events. **A** Schematic representation of plasmid fusion-mediated population rescue under antibiotic selection. Fusion between a conjugative plasmid (pC) and a non-transmissible plasmid (pNT) generates a multireplicon plasmid capable of transfer and survival under multidrug selection. Red triangles indicate homologous regions mediating plasmid fusion. **B** Conjugation assays selecting either for transfer of the conjugative plasmid alone (black) or for fused plasmids carrying both plasmid markers (purple). The *x*-axis indicates plasmids present in donor strains and the *y*-axis shows conjugation efficiency. Data represent mean values from *n* = 3 biological replicates; individual data points are shown. **C** Secondary conjugation assays using candidate fusion plasmids isolated in (**B**) as donors into a fresh recipient strain. Selection was performed for markers associated with both the conjugative and non-transmissible plasmids. Data represent mean values from *n* = 3 biological replicates; individual data points are shown. **D** Representative plasmid fusion events resolved by long-read sequencing. Gtm gentamicin resistance, Cd cadmium resistance, Tmp trimethoprim resistance, Bla β-lactam resistance.

We hypothesised that strong antibiotic pressure could provide such selection, by favouring fusion between a conjugative plasmid and a non-transmissible plasmid and thereby rescuing bacterial populations under conditions that neither plasmid alone could withstand. To test this, we designed an experimental system in which a donor strain carrying both a large conjugative plasmid (pGO1, p.EvoF or p.731) and a smaller non-transmissible plasmid (p.Evo, p.144 or p.3912) was co-cultured with a recipient strain bearing a distinct antibiotic resistance marker. Under these conditions, survival under three-antibiotic selection could only occur through plasmid fusion followed by conjugative transfer (Fig. 3A).

Cultures were plated under two selection regimes: two antibiotics, to measure baseline conjugation efficiency, and three antibiotics, to specifically select for fused plasmids. Under two-antibiotic conditions, conjugation efficiencies were high, yielding 10^4^–10^6^ transconjugants per mL for pGO1, p.EvoF and p.731 (Fig. 3B). In contrast, only a small number of colonies were recovered under three-antibiotic selection. To determine whether these colonies carried fused plasmids, three isolates from each condition were used as donors in secondary conjugation assays into a fresh recipient strain. In all cases, conjugation efficiencies returned to levels comparable to those of the parental conjugative plasmids (10^3^–10^6^ transconjugants per mL; Fig. 3C), suggesting that the observed low frequencies were due to low fusion rates.

Long-read sequencing of these isolates confirmed that they carried fused plasmids containing both the conjugative and non-transmissible elements (Figs. 3D and S3C). Because fusion events occur prior to antibiotic selection (Fig. S3B), the reduction in transfer efficiency between two- and three-antibiotic conditions provides an estimate of fusion frequency in the population. Across all tested combinations, fusion events were 3–4 orders of magnitude less frequent than standard conjugation, confirming that plasmid fusion is rare under baseline conditions (Fig. 3B). Short-read sequencing of plasmids before and after fusion showed that Rep13, which allows a high copy number, is ultimately detected at a level similar to that of the large conjugative plasmid after fusion, suggesting regulatory interactions between replication systems that favour the dominance of lower-copy-number replicons in multireplicon plasmids (Supplementary Data 6).

Together, these results demonstrate that although plasmid fusion occurs infrequently, strong antibiotic pressure can selectively enrich fused plasmids. By conferring conjugative mobility to previously non-transmissible elements, fusion enables population survival under multidrug stress and provides a selective route by which rare fusion events can rise to prominence. This selective enrichment offers a mechanistic explanation for the widespread prevalence of multireplicon plasmids observed in genomic databases.

### Homologous recombination and transposition shape plasmid fusion

Plasmid fusion can arise through multiple molecular mechanisms, including homologous recombination (HR) and transposition, both of which can contribute to plasmid diversification. Because transposition creates large repeats that can favour HR, IS can result in both HR and transposition. To define the role of IS*257* in mediating fusion, we analysed long-read sequencing data from fused plasmids isolated during the antibiotic selection experiments (Figs. 3D and S3C, D). In all sequenced clones, IS*257* elements flanked the fusion junctions, indicating that fusion occurred either through HR between IS*257* copies or via IS*257*-mediated transposition.

To distinguish between these mechanisms, we examined fusion junctions for signatures of transposition, specifically the presence of 7-bp direct repeats characteristic of IS*6*-family transposase activity^28^. In two clones (TI226 and TI228), fusion junctions were flanked by newly generated IS*257* copies and 7-bp direct repeats (Figs. 3D and S3C), consistent with transposition events. These insertions occurred at multiple sites within the small non-transmissible plasmid p.3912 and were independent of its native IS*257* element (Fig. S3D). In contrast, four clones (TI564, TI243, TI246 and TI249) lacked direct repeats, and fusion occurred directly between pre-existing IS*257* elements on each plasmid, consistent with HR-mediated co-integration (Fig. S3C).

To experimentally test whether both HR and transposition can independently mediate plasmid fusion, we constructed derivatives of p.3912 lacking IS*257* (p.noIS), as well as a variant containing an alternative region of homology (p.HR, containing the trimethoprim resistance gene shared with p.EvoF and p.731) (Fig. S4A). Under antibiotic pressure, all plasmid variants supported population rescue following conjugation with p.731 and p.EvoF (Fig. S4B). Long-read sequencing confirmed plasmid fusion in all cases and revealed junction architectures consistent with either HR or transposition (Fig. S4C and E). For p.noIS, which does not contain any homologous sequence or IS genes, transposition can still be mediated by the IS*6* element carried on the conjugative plasmid. In the case of p.HR, both homologous recombination and transposition can be observed (Fig. S4E). Analysis of transposition-derived junctions identified a conserved 7-bp direct repeat motif, allowing definition of a consensus insertion site (TTTWTAD) (Fig. S4D).

Together, these results demonstrate that plasmid fusion can be mediated by both homologous recombination and transposition. Moreover, short regions of sequence homology (> 600 bp), including conserved replication genes, resistance determinants or transposase sequences, are sufficient to promote co-integration. This mechanistic flexibility provides a basis for the widespread generation of plasmid diversity and illustrates how mobile genetic elements facilitate rapid adaptation under selective pressures such as antibiotic exposure.

### Increased plasmid size does not consistently impose a major fitness cost after fusion

Although plasmid fusion is frequent and widely represented in genomic databases, very few *Staphylococcus* plasmids exceed 45 kb, even among those with clear fusion signatures (Fig. 1B). Notably, 45 kb corresponds to the maximum packaging capacity of most *Staphylococcus* transducing phages^10^, supporting the idea that phage-mediated transfer imposes an upper limit on plasmid size^11^. We considered three non-mutually exclusive explanations for this pattern: (i) a fitness cost associated with carrying large plasmids, (ii) resolution of fused plasmids after co-integration and (iii) selection for deletions, potentially driven by phage-mediated transfer constraints.

**Fig. 4:**
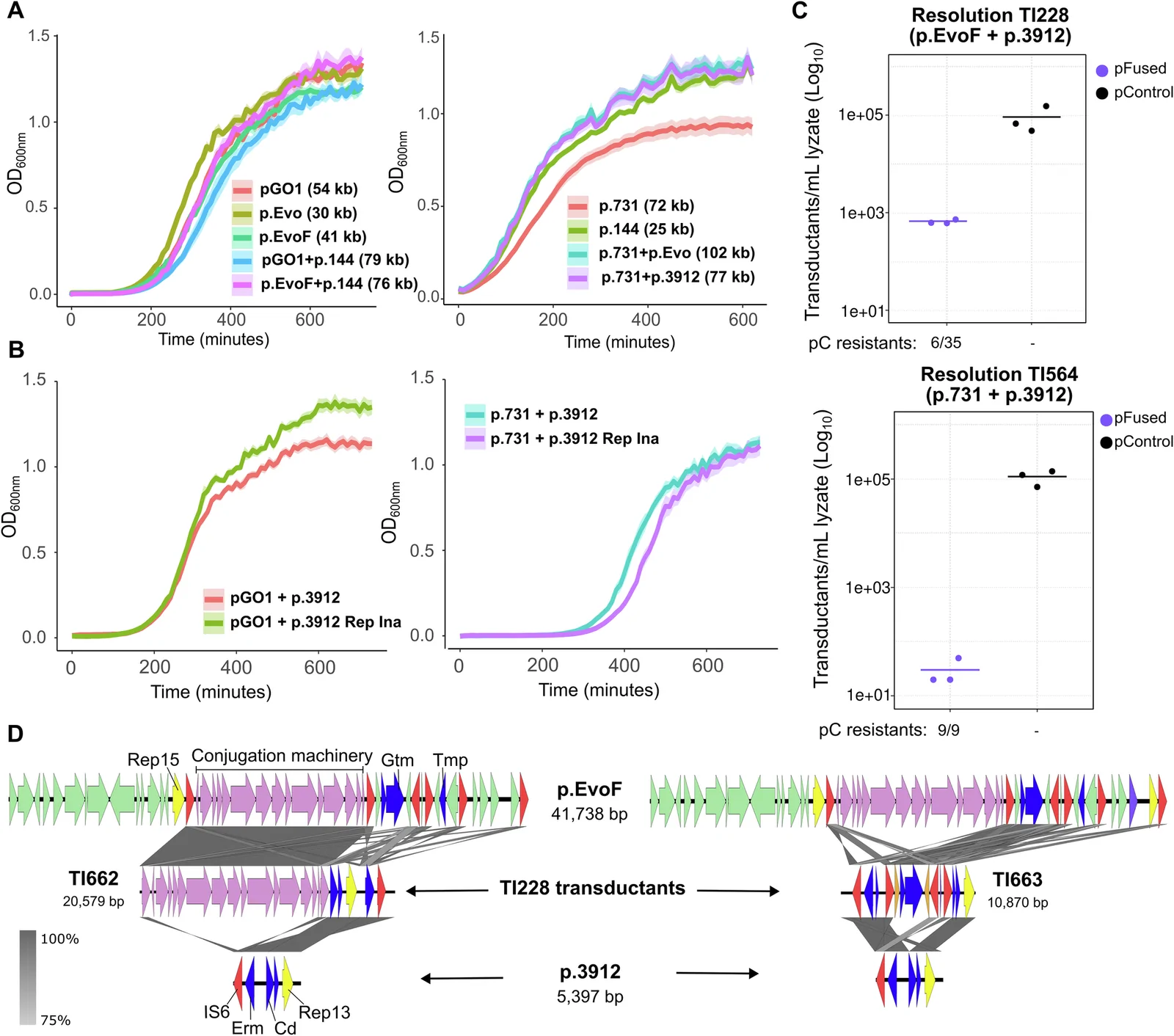
Fused plasmids undergo resolution and deletion without major fitness costs. **A** Growth curves of RN4220 strains carrying fused plasmids or their parental derivatives generated from pGO1 (left) or p.731 (right) backgrounds. Growth was monitored by OD600 measurements over 12 h (*n* = 4). Shadows in growth curves indicate standard error. **B** Growth curves of RN4220 strains carrying fused plasmids with either one or two functional replication systems. Growth was monitored by OD600 measurements over 12 h (*n* = 5). Shadows in growth curves indicate standard error. **C** Resolution assay using phage 80α-mediated transduction. Transduction efficiencies of fused or unfused plasmids were determined by selecting for the p.3912 erythromycin-resistance marker. Purple symbols represent fused plasmids and black symbols represent unfused controls. Because fused plasmids exceed the packaging capacity of phage 80α (~ 41 ± 2.5 kb), recovery of erythromycin-resistant colonies reflects plasmid resolution or deletion events. Data represent mean values from *n* = 3 biological replicates; individual data points are shown. **D** Representative deletion derivatives recovered following the resolution assay and characterized by long-read sequencing.

To test whether increased plasmid size or the presence of multiple replication systems imposes a fitness cost, we compared the growth of bacteria carrying fused plasmids with that of strains carrying their unfused parental plasmids. Using fused plasmids generated in our experimental system, we first assessed whether plasmid size correlated with reduced growth (Fig. 4A). No consistent size-dependent fitness cost was detected. The 76-kb fused plasmid derived from p.EvoF grew comparably to its parental plasmids pGO1 (54 kb) and p.EvoF (42 kb). Similarly, in the p.731 background, fused plasmids of 77 and 102 kb grew as well as, or slightly better than, the smaller p.144 (26 kb) and the original p.731 plasmid, which curiously showed the poorest growth. Only the 79-kb fused plasmid derived from pGO1 showed a modest fitness cost, with reduced total growth and a longer lag phase. The smallest derivative, p.Evo, displayed slightly faster growth, likely reflecting the absence of conjugation machinery as observed in a *K. pneumoniae* plasmid^40^.

We next examined whether carrying two active replication systems within a single plasmid affected bacterial fitness. We compared growth of strains harbouring fused plasmids in which one Rep gene was naturally inactivated during fusion with those retaining two functional Rep genes (Fig. 4B). No consistent growth differences were observed: in one case, pGO1 carrying two active Rep genes showed a slight growth reduction, whereas in another, p.731 with two active Rep genes displayed a modest fitness advantage.

Together, these results indicate that neither increased plasmid size nor the presence of multiple active replication systems consistently imposed a major fitness cost under the conditions tested, although modest effects were observed in some plasmid backgrounds. The predominance of smaller plasmids in natural populations, therefore, cannot be explained by fitness limitations alone, implicating alternative selective forces, such as deletion processes or phage-mediated constraints, in maintaining compact plasmid sizes.

### Resolution and deletion diversify fused plasmids

Previous studies have shown that plasmids generated by transposition can sometimes resolve back into their original components^28,41,42^. To determine the intrinsic fates of fused plasmids after co-integration, we examined the outcomes of fusion events independently of their long-term selective success.

Using fused derivatives of pGO1–p.3912 and p.731–p.3912 generated in earlier experiments, we exploited 80α phage-mediated transduction and selected for resistance encoded by the smaller plasmid. Because large, fused plasmids exceed the DNA packaging capacity of most staphylococcal phages, colonies recovered under these conditions were expected to reveal post-fusion processing events, including resolution or deletion.

Analysis of recovered colonies revealed two distinct mechanistic outcomes. In two sequenced clones (TI267, TI273), the original small plasmid was regenerated, indicating resolution of the co-integrated plasmid back into its parental components. In parallel, a second class of colonies retained resistance markers associated with the large conjugative plasmid. Among 35 colonies examined for pGO1–p.3912 and 9 for p.731–p.3912, 6 and 9 colonies, respectively, displayed this phenotype (Fig. 4C).

Long-read sequencing showed that these latter isolates carried novel intermediate-sized plasmids generated by deletion rather than simple resolution (Fig. 4D). In one case, deletion of the pGO1 backbone resulted in a p.3912 replicon plasmid (Rep13) that contained the full pGO1 conjugation machinery, effectively converting the original non-transmissible Rep13 plasmid sub-family into a conjugative plasmid. In all sequenced cases, deletion junctions were consistent with homologous recombination between IS*257* elements (Fig. 4D).

Deletion events were also detected in the absence of phage infection. Using plasmids carrying multiple IS*257* copies, we amplified and sequenced recombination scars consistent with IS*257*-mediated deletions occurring naturally at the population level (Fig. S5).

Together, these results demonstrate that fused plasmids can act as diversification intermediates from which multiple evolutionary outcomes arise, including resolution back to parental plasmids and deletion-driven formation of novel chimeric elements.

### Phage pressure selectively enriches small plasmid derivatives

While fused plasmids generate multiple derivative forms, the relative abundance of these outcomes in natural populations is expected to depend on natural selection. In particular, bacteriophages impose strong physical constraints on DNA packaging, potentially favouring smaller plasmid variants capable of being transduced.

To test whether phage pressure selectively enriches specific post-fusion outcomes, strains carrying multireplicon plasmids larger than 45 kb were infected with phages 80α or ϕ2339 and transduction experiments were performed into a new recipient strain. Instead of selecting for the resistance marker of the small plasmid, as done previously to detect plasmid resolution, we applied selection for the marker present on the large conjugative plasmid or for both markers, in order to select for smaller plasmid variants carrying different combinations of AMR genes. We predicted that phage-mediated transfer would act as a filter, enriching for plasmid variants compatible with phage packaging carrying various combinations of genes as observed previously (Fig. 4D).

**Fig. 5:**
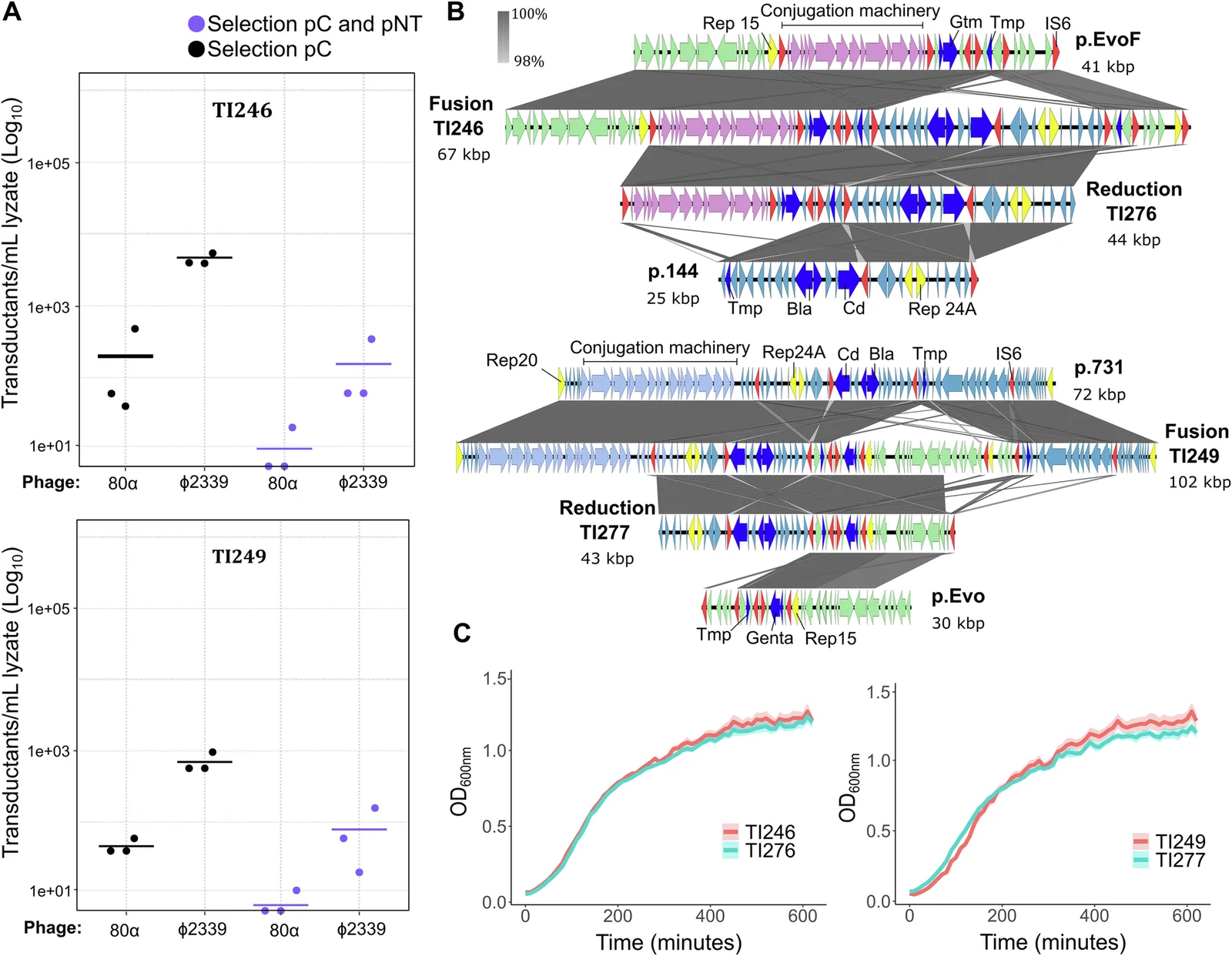
Phage-mediated selection enriches intermediate-size plasmid derivatives. **A** Transduction assays using phages 80α or Φ2339 on strains carrying fused plasmids. Black symbols indicate selection for the conjugative plasmid marker alone, whereas purple symbols indicate selection for markers from both plasmids. Because fused plasmids exceed phage packaging limits, transduction enriches smaller plasmid derivatives. Data represent mean values from *n* = 3 biological replicates; individual data points are shown. **B** Sequence alignments of representative plasmid derivatives recovered following phage transduction. **C** Growth curves of RN4220 strains carrying fused plasmids or representative smaller derivatives recovered after transduction. Growth was monitored by OD600 measurements over 12 h (*n* = 3). Shadows in growth curves indicate standard error.

Consistent with this prediction, transduction recovered a diverse spectrum of plasmid derivatives in all plasmid–phage combinations tested (Fig. 5A, B). Long-read sequencing revealed plasmids of intermediate sizes (33.5–44.5 kb) with distinct architectures, including deletion derivatives and novel combinations of resistance genes and conjugation modules (Figs. 5 and S6).

Importantly, growth assays showed no detectable fitness cost associated with these smaller derivatives relative to their fused precursors (Fig. S6), indicating that their enrichment was driven by phage selection rather than growth advantage.

Together, these results support the idea that bacteriophage pressure does not generate plasmid diversity de novo, but can selectively enrich specific outcomes generated by plasmid fusion, favouring smaller, transducible variants. This selective filtering explains how plasmid size remains constrained in nature while preserving extensive genetic diversity.

## Discussion

**Fig. 6:**
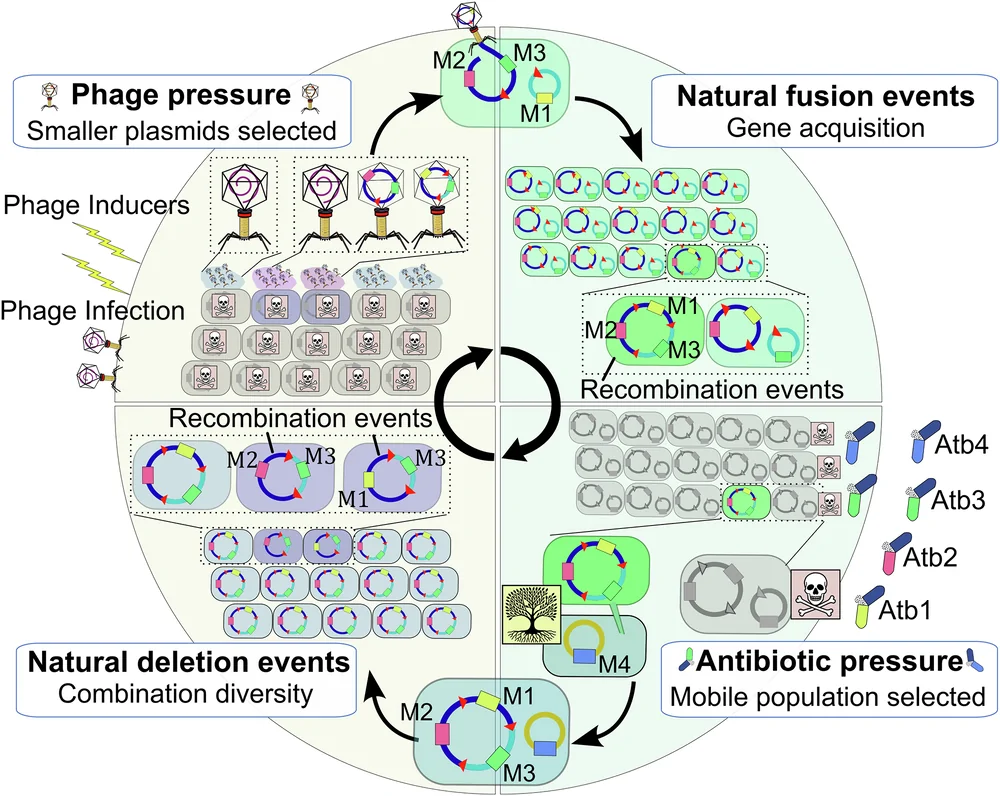
Proposed model for plasmid diversification through fusion and deletion cycles. Rare fusion events mediated by homologous recombination or transposition generate multireplicon plasmids with expanded gene repertoires and transfer potential. Under antibiotic selection, these fused plasmids can be enriched within bacterial populations. Subsequent deletion or resolution events generate smaller plasmid derivatives carrying distinct combinations of accessory genes and mobility functions. Phage-mediated transfer selectively favours plasmids compatible with phage packaging constraints, enriching smaller transmissible variants. Together, these processes generate dynamic plasmid populations containing conserved backbone functions and diverse accessory modules.

Plasmids are central engines of bacterial adaptation, yet the evolutionary forces shaping their diversity remain poorly understood. Integrating our bioinformatic, genetic and experimental data, we identify a plasmid life cycle that operates at the population level and is driven by mobile genetic elements. In *S. aureus*, this life cycle is characterised by alternating phases of fusion, diversification and ecological selection (Fig. 6). Under antibiotic pressure, rare fusion events generate multireplicon plasmids that merge genetic repertoires and mobilize previously non-transmissible plasmids, potentially rescuing bacterial populations under strong antibiotic stress. While multireplicon plasmids resulting from plasmid fusion are common in nature (~ 50%), only 6% of plasmids are larger than 45 kb in *Staphylococcus*. This suggests that plasmid fusions are accompanied either by fission or gene deletion events (or both) that drive plasmid diversification. They can resolve into parental components or undergo deletions to generate intermediate-sized chimeric plasmids that retain key functional modules, producing a repertoire of derivatives from a single large-fused plasmid ancestor. Although size-associated fitness costs and plasmid instability have previously been proposed as important drivers of plasmid selection^43–45^, our results indicate that phage-mediated transfer can act as a strong selective filter on plasmid size. During bacterial lysis, phages selectively enrich smaller plasmids by permitting transfer only of intermediate-sized plasmids compatible with generalized transduction. Together, this fusion-diversification-selection process explains how plasmid populations maintain conserved backbones, constrained size distributions and extensive accessory gene diversity without preserving strict plasmid identity.

ISs emerge as central catalysts of plasmid diversification. They facilitate both fusion and deletion through homologous recombination and transposition, enabling rapid structural rearrangements across short regions of homology. This role is consistent with observations in various bacterial species, where IS*6*-family elements mediate plasmid co-integration^17,21,29,33^ or plasmid deletion^43^ via similar mechanisms. Importantly, IS-mediated rearrangements do not merely generate transient intermediates but can give rise to stable plasmid variants that persist under selection. Conjugative plasmids display particularly high fusion frequencies, mirroring patterns reported in *E. coli* and other species where IS density is elevated near AMR genes^27,46,47^. Beyond IS elements, other conserved genetic features, including resistance cassettes (Fig. S4) and replication genes, can also serve as substrates for homologous recombination. While replication genes represent potential recombination targets, plasmid incompatibility limits the stable coexistence of identical replicons within the same cell^48^, suggesting that plasmid fusion is shaped by a balance between recombination potential and incompatibility constraints.

Although multireplicon plasmids have been reported for decades, they were long regarded as rare or exceptional products of plasmid evolution, often described as isolated curiosities rather than as evolutionarily consequential entities. Recent population-scale analyses, particularly in *Enterobacteriaceae*, have challenged this view by revealing a historical expansion of multireplicon plasmids during the antibiotic era, suggesting that they may play a major role in contemporary resistance evolution^23,24,26^. However, these studies did not determine precisely how frequently plasmid fusion occurs, how such rare events become evolutionarily significant, or whether multireplicon plasmids represent stable endpoints or transient stages within broader evolutionary dynamics. Our results directly address these gaps by showing that multireplicon plasmids are common in *Staphylococcus*, arising repeatedly through MGE-driven fusion that produces large plasmids functioning as key intermediates in a dynamic evolutionary process. Plasmid fusion generates large multireplicon plasmids that enhance genetic content and provide plasmid mobility. In *Staphylococcus*, multireplicon plasmids are widespread in genomic databases, representing nearly half of all plasmids analyzed, but we showed that large plasmids are rare in nature (6% of *Staphylococcus* plasmids > 45 kb). This apparent paradox can be explained by the branching evolutionary trajectories of large multireplicon plasmids following plasmid fusion and subsequent selection (Figs. 3 and 6). After fusion, plasmids can resolve into their original components or undergo deletion to generate novel chimeric plasmids retaining key functional modules. These branching outcomes substantially increase accessory gene diversity within bacterial populations, generating a spectrum of plasmid architectures from a single large multireplicon plasmid.

Environmental pressures then determine which of these variants persist. Fitness cost has often been invoked as the primary driver of plasmid deletion selection in various bacterial species^44,49–51^. In our case, we show that the individual effect of plasmid size on *S. aureus* fitness is weak. In contrast, our results support the idea that bacteriophage predation can impose a strong physical constraint on plasmid size by selectively enriching variants compatible with phage-mediated transduction. Large fused plasmids are unable to be fully packaged and are lost, whereas deletion derivatives and resolved plasmids that fall below phage packaging limits are preferentially propagated. This size-dependent filtering is further reinforced by staphylococcal pathogenicity islands, which package only small DNA molecules, thereby favouring even more compact plasmid architectures^11^.

Altogether, our results provide mechanistic insight into plasmid evolution: antibiotics select for plasmid fusion events that combine multiple resistance determinants, while efficient transfer, as demonstrated here for phages, constrains plasmid size by selecting deletion-derived variants. A defining feature of the plasmid life cycle described here is that it relaxes, rather than enforces, the conservation of genetic architecture across generations. In contrast to many MGEs, whose life cycles tend to preserve a core architecture, it prioritizes diversification over replication fidelity. Each fusion event seeds multiple evolutionary trajectories, producing a branching set of descendants rather than a single reverting form. This controlled diversification is not a limitation of the system but its principal evolutionary advantage, allowing plasmid populations to explore a wider genetic space while remaining constrained by transmission ecology. We propose that this highly diversifying, MGE-driven life cycle represents a general principle of plasmid evolution, particularly in species where multiple mobile elements coexist and interact. This process shapes the mosaic architecture of modern resistance plasmids and aligns with recent work showing that population-level diversity enhances survival under fluctuating antibiotic and phage pressures^52^.

An important layer not explicitly incorporated into our model is the role of plasmid-encoded phage defence systems. Defence mechanisms such as restriction–modification systems or abortive infection modules may influence which plasmid variants persist following fusion or deletion. Large conjugative plasmids combining AMR with effective phage defence could be preferentially retained. Conversely, smaller plasmids may escape restriction barriers more efficiently^53,54^ and achieve higher resistance expression due to increased copy number^55^. Incorporating phage defense systems into future models will further refine how plasmid mobility, gene content and architecture are optimized under complex ecological pressures. Additionally, the reduced copy number observed in multireplicon plasmids points to the existence of intriguing inter-replicon regulatory systems that results in plasmid copy numbers lower than those of the highest copy-number plasmid. This warrants further investigation since it may shape the fate of fusion events and modulate plasmid incompatibility in multireplicon backgrounds.

Although this study focuses on *S. aureus*, the underlying principles are likely generalisable. Multireplicon plasmids, IS-mediated fusion and deletion events are reported in Enterobacteriaceae, *Klebsiella*, *Salmonella*, *Acinetobacter* and *Citrobacter*^13,16,18–20,26,56^, often with major consequences for plasmid mobility and resistance dissemination^57^. Plasmids may therefore represent an extreme case of evolvable mobile elements, in which repeated interactions with other MGEs amplify genetic diversity beyond what is observed in more canonical life cycles.

We conclude that plasmid diversification can emerge from a dynamic evolutionary cycle driven by mobile genetic elements and ecological selection. By framing plasmid evolution as a cyclical, population-level process rather than the persistence of individual elements, our model explains the rapid emergence, stability, and global dissemination of multidrug-resistant plasmids. This conceptualization provides a framework for anticipating resistance evolution and highlights the importance of considering phage-plasmid interactions when designing antimicrobial intervention strategies, including phage therapy.

## Methods

### Oligonucleotides and plasmids

All oligonucleotides used in this study were supplied by Sigma and are listed in Supplementary Data 5-A. All plasmids constructed and used in this study are listed in Supplementary Data 5-B.

### Bacterial strains and growth conditions

The bacterial strains used in this study are listed in Supplementary Data 5-C. For all experiments, *S. aureus* was grown in Tryptic Soy Broth (TSB) at 37 °C with shaking (180 rpm) overnight until stationary phase. For enumeration of colony-forming units (CFU) on solid media, cultures were plated on Tryptic Soy Agar (TSA) and incubated at 37 °C. When required, the media was supplemented with the following antibiotic concentrations: gentamicin 10 µg/ml; erythromycin 10 µg/ml; trimethoprim 20 µg/ml; chloramphenicol 10 µg/ml; CdCl₂ 100 µM; tetracycline 3 µg/ml; rifampicin 10 µg/ml.

When using *Saccharomyces cerevisiae* (provided by Dr. Carole Lartigue, INRAE) as a cloning platform, *S. cerevisiae* VL6-48N was grown in YPDA broth (Takara, 630464) overnight at 30 °C with shaking (200 rpm). When required, minimal SD medium (SD-Trp broth; Takara, 630411 and 630413) was used at 30 °C with shaking at 220 rpm.

### Bioinformatic analyses

3076 *Staphylococcus* plasmids were downloaded from PlasmidScope database^58^ (version 2024-12-04, all the plasmids were collected from PLSDB^59^). After removing potential chromosomal contigs and merging identical sequences, 2564 non-redundant complete plasmids in a size range of 2–170 kb were kept for the further analysis (Supplementary Data 1).

Plasmid mobility typing was performed using MacSyFinder v2.1.4^60^ with CONJScan^61^ model v2.0.1. OriT sequences were searched using BLASTn^62^ (v 2.17.0 with parameter -task blastn-short, identity > 80% and coverage > 80%) with 117 experimentally valid oriT sequences from OriTDB v2.0^63^, the best hit from overlapping hits was kept. Plasmid mobility types were classfied into plasmid encoding oriT sequence (pOriT), plasmid encoding relaxase (pMOB), conjugative plasmid (pCONJ), decayed conjugative plasmid (pdCONJ) and plasmid lacking mobility (pNT). 23 plasmids with mobility type “pdCONJ” are grouped into “pMOB” in the subsequent analysis.

Plasmid replicon typing was performed using PlasmidFinder v2.1.1^64^ and replicon database v2.2.0 (with parameters -t 0.8 -l 0.6, identity > 80% and coverage > 60%), the best hit from overlapping hits was kept (Supplementary Data 2). Replicon identification was sequence-based, and the detection of a replicon was not taken as evidence that the corresponding replication system was functionally active.

Replicon co-occurrence networks were plotted using Cytoscape v3.10.3^65^.

Insertion sequence IS*6* and IS*Bli29* were detected using digIS v1.2^66^ (Supplementary Data 3) and IS*6* transposase sequences were cross validated using Blastn with a pre-build blast database of Tncentral^67^ and ISFinder^68^ (version 2025-05-16), for each query the best hit was kept (Supplementary Data 4).

### Detection of deletion and fusion event in *Staphylococcus*

Bacterial strain *S. aureus* RN4220 containing one plasmid or combinations of plasmids was cultured overnight, and plasmids were extracted from 1 mL of culture using the ZR BAC DNA Miniprep Kit (Zymo Research, D4049). PCRs were performed using Advantage II HF polymerase (Takara), following the manufacturer’s recommendations.

### Large plasmid construction

The natural plasmid pGO1 lacking the conjugative machinery and containing a chloramphenicol resistance gene was constructed in the non-commercial *Saccharomyces cerevisiae* VL6-48N strain, as previously described in refs. ^69–71^ with several modifications. Overlapping PCR products and YAC (200 ng of each PCR product and 100 ng of linearized YAC in 10 µL water) were co-transformed into VL6-48N. Individual yeast colonies were picked and streaked onto SD-Trp plates, incubated for 2 days at 30 °C, and then patched onto fresh SD-Trp plates and incubated for another 2 days at 30 °C. Total genomic DNA was extracted from yeast transformants according to ref.^69^. PCRs were performed using Advantage II HF polymerase (Takara), following the manufacturer’s recommendations. 1.7 kb DNA scars remain on the plasmid and include YAC elements for yeast replication and selection.

### Small plasmid construction

Small p.3912 derivatives lacking IS257, or in which IS257 was replaced by a sequence allowing homologous recombination with pGO1 and p.731, and carrying the trimethoprim resistance gene, were constructed in *S. aureus* RN4220. Overlapping PCR fragments were generated and assembled using the HiFi DNA Assembly Kit (NEB). The reaction products were dialyzed on a membrane filter for 5 min, and the remaining mixture was transformed into *S. aureus* RN4220 (2.2 kV, 100 Ω, 2 mm) and plated on TSA containing erythromycin. Colonies were then analyzed, and plasmids were sequenced.

### Bacterial growth curves

Growth kinetics were assessed using *S. aureus* RN4220 derivatives carrying the indicated plasmids. Overnight cultures were inoculated into fresh TSB at a 1:100 dilution and grown at 37 °C with shaking (180 rpm). Cultures were transferred to 96-well flat-bottom plates (200 μL per well; three technical replicates per strain), and optical density at 600 nm (OD600) was measured every 10 min for 24 h using a microplate reader with continuous orbital shaking between reads.

### Phage infection

Bacterial cultures containing the appropriate plasmid were grown to an OD600 of 0.15. One milliliter of culture was mixed with 100 μL of phages supplemented with 5 mM CaCl₂ and incubated at 30 °C for 3 h, followed by overnight incubation at room temperature. The resulting phage lysate was filtered to remove remaining bacterial cells.

### Phage titer

*S. aureus* RN4220 was grown overnight, then subcultured at a 1:50 dilution into fresh TSB and grown to an OD600 of 0.2. Bacterial lawns were prepared by mixing 100 μL of cells with phage top agar (supplemented with CaCl₂ to a final concentration of 5 mM) and pouring the mixture onto PBA plates supplemented with 5 mM CaCl₂. Serial dilutions of phage lysates were prepared in phage buffer (50 mM Tris, pH 8; 1 mM MgSO₄; 4 mM CaCl₂; 100 mM NaCl) and spotted onto the bacterial lawns, which were then incubated overnight at 37 °C. Plaques were counted and used to quantify the phage produced during infection.

### Plasmid transduction

*S. aureus* RN4220 was grown in TSB to an OD600 of 1.4. For transduction experiments, 100 μL of phage lysates were added to each culture along with 5 mM CaCl₂. The cultures were incubated statically at 37 °C for 30 min and then plated onto TSA plates containing 17 mM sodium citrate and the appropriate antibiotic for selection of the expected plasmid. Plates were incubated for 24–48 h, and colonies were counted. The transfer efficiency (TE) per donor cell was calculated as the number of Transductant Units (TrU) per mL of lysate.

### Conjugation assays

Conjugation assays were performed using mixed cultures of the donor strain (pGO1, pGO1EvolF, pWBG731, TI226, TI228, TI232, TI233, TI243, TI246, TI249, TI538, TI541, TI542, TI545, TI558, TI564, TI635, TI636, TI638, TI639, TI641 and TI642) and the recipients (RN4220 strains resistant to Tet or Rif antibiotics). Overnight cultures of donor and recipient cells were washed with PBS to remove antibiotics and resuspended in TSB without antibiotics. Equal numbers of donor and recipient cells (5 × 10^7^ CFU) were combined. The entire mixture was spotted onto a sterile 0.45 μm pore nitrocellulose filter (MF-Millipore™ Membrane Filter) placed onto TSA. The mixture was allowed to dry onto the filter before incubating at 37 °C for 24 h. Donor and recipient only controls were included in parallel. Following incubation, filters were transferred to microtubes and vortexed vigorously in 1 mL phosphate buffered saline to detach the bacteria from the filters for quantification. To determine plasmid transfer frequencies, donors and recipients were titrated on TSA and TSA containing the appropriate selective antibiotic. Transfer efficiency was calculated as number of transconjugants divided by the total number of recipients.

### Plasmid sequencing

Plasmid architectures were determined by long-read sequencing performed by Plasmidsaurus (San Francisco, CA, USA) using Oxford Nanopore Technologies. Mean read depth was approximately 100×. FASTA files for the sequenced plasmids are listed in Supplementary Data 5. Illumina whole-genome sequencing reads were provided by SeqCenter (Pittsburgh, USA). The coverage per position was determined using samtools depth v1.19. Raw data are listed in Supplementary Data 6. The copy number was obtained as the mean coverage of the plasmid divided by the average coverage of the chromosome. Sanger sequencing of recombination scars was performed using Eurofins Genomics.

### Statistical analyses

The enrichment of different replicon families in multiple-/single-replicon plasmids was tested using Fisher’s exact test in contingency analysis and correction for multiple comparisons with false discovery rate^72^. The probability of a plasmid being multireplicon rather than single-replicon  was modeled using a binomial generalized linear model using the glm function (R package stats v4.4.3, with parameter family = binomial (link = “logit”)). The effects of plasmid size (log10 transformed), IS_presence (plasmid encoding transposase of IS*6*/ IS*Bli29* or not), and plasmid mobility (mobile (i.e., pOriT, pMOB, pdCONJ, pCONJ) or non-mobile (pNT)) were tested. All statistical analysis were conducted in R v4.4.3.

### Reporting summary

Further information on research design is available in the Nature Portfolio Reporting Summary linked to this article.

## Supplementary information

Supplementary Information

Description of Additional Supplementary Files

Supplementary Data 1

Supplementary Data 2

Supplementary Data 3

Supplementary Data 4

Supplementary Data 5

Supplementary Data 6

Reporting Summary

Transparent Peer Review file

## Source data

Source Data

## Data Availability

The genomics maps generated in this study are accessible in Supplementary Data 5. The experimental data generated in this study are provided in the Source data file. Source data are provided with this paper.
