# Supplementary material for: Mobile genetic elements drive a plasmid fusion and deletion lifecycle shaping evolution and antimicrobial resistance
Source: Nat Commun. 2026 Sep 15;17:10849. doi: 10.1038/s41467-026-77678-8 (PMC13672794; doi:10.1038/s41467-026-77678-8)
Supplement: Supplementary file 1 [file 41467_2026_77678_MOESM10_ESM.pdf]

nature portfolio

Peer Review File

Mobile genetic elements drive a plasmid fusion and deletion lifecycle shaping evolution and antimicrobial resistance

Corresponding Author: Professor José Penadés

This file contains all reviewer reports in order by version, followed by all author rebuttals in order by version.

Version 0:

Reviewer comments:

Reviewer #1

(Remarks to the Author)
The authors present a study on plasmid evolution in Staphylococcus aureus, proposing that mobile genetic elements (MGEs) drive a cyclical fusion–deletion process that shapes plasmid diversity. The work combines large-scale genomic analyses with experimental work to support a model in which MGE-mediated fusion events generate plasmids with expanded gene content, while opposing selective pressures favour deletion derivatives. Overall, the study addresses an important question in plasmid evolution and provides a compelling conceptual framework.

My area of expertise is bacterial genetics, and therefore my review focuses primarily on this aspect.

1) Line 119. In the analysis used to define a plasmid as multireplicon, did you consider not only the presence of multiple rep genes but also their associated origins of replication (ori)?

2) Line 187. The text refers to Figure 2C, but if I am not mistaken, it should refer to Figure 2B.

3) Line 188. The 6% of plasmids larger than 45 kb, are they using the same ori as the smaller plasmids, or do they represent a distinct group?

4) Lines 230–231. The Materials and Methods section does not describe how the conjugation experiments were performed (solid or liquid mating, incubation time, donor:recipient ratio, etc.). In addition, the way transconjugants are presented (Figures 3B, 5A, S3B, S4B) is not standard in the literature. Several calculation methods can be used (some accounting for donor numbers and mating time), but the simplest is to express results as the number of transconjugants divided by the total number of recipients. As currently presented, the data do not provide clear information about transfer frequencies.

5) Regarding the Materials and Methods, some plasmids were constructed by yeast-mediated assembly, but it is unclear how the YAC backbone was removed from the final plasmid. Please clarify.

6) Figure S3C should be moved to the main figures and presented together with Figure 3B.

7) Figures 3D and S3D should be combined into a single figure. Figure S3D also needs to be better organised, and the legend should be clarified.

8) Figure legends in general need improvement. For example, avoid false friends such as “experience,” and provide sufficient detail so that the reader does not need to infer key information. The legends should be more self-explanatory.

9) Lines 243–245. This is an extremely interesting observation that is not discussed at all by the authors. If rep13 (a high-copy-number replicon, as described by the authors) is not mutated, this suggests some level of regulation between replication systems in fused plasmids. One might expect the copy number of a fused plasmid to reflect that of the highest-copy replicon present; however, this is not observed (figure S6). Instead, plasmids containing a high-copy-number rep display a low copy number overall. This strongly suggests regulatory interactions between replicons, possibly involving dominance or recessiveness effects. This point is highly interesting and deserves discussion and, in my opinion, future research.

10) Line 266. Why is Figure S4C introduced before Figures S4A and S4B?

11) Line 273. Introduce the name of plasmid pHR.

12) Line 273. Selective conditions do not favour fusion per se; rather, they select for fusion events that have already occurred.

13) Line 278. This should refer to Figure S4E, not S4C–S4D.

14) Line 280. Define what is meant by "short."

15) Line 298. According to the figure, the plasmid is 79 kb. Otherwise, the 76 kb plasmid corresponds to the pEVO derivative.

16) Line 299. The blue line (pGO1 + p144) indicates a fitness cost, with reduced total growth and a longer lag phase.

17) Line 302. The plasmid is 77 kb according to the figure.

18) Line 308. There are differences in pGO1: the version carrying two rep genes (red line) shows reduced total growth.

19) Line 323. Remove the reference to Figure 4C.

20) Line 342. This point has already been demonstrated in the previous section.

21) Line 371. Remove "transiently." The experiments performed do not constitute evolutionary assays, and therefore the long-term fate of newly generated plasmids is unknown.

22) Line 373. "These fused plasmids do not represent…" This statement should either be made more speculative or supported with additional evidence. Fused plasmids can be found in databases; for example, pWBG731 carries two functional rep genes. Without direct evolutionary analysis or population studies, it is not possible to conclude that these fused plasmids do not persist.

23) Line 376. The authors place strong emphasis on phage-mediated transduction, but quantitative data are needed to support this claim. Are there active mechanisms promoting phage encapsidation of these plasmids (e.g., pac or pac-like sites), or does transfer occur via generalized transduction? What proportion of capsids carry plasmid DNA?

Capsids may contain: phage genomes, SaPIs (if present in the strain), DNA derived from lateral transduction, chromosomal DNA via generalized transduction or plasmid DNA.
So, unless otherwise proven, the observed plasmid sizes could reflect additional factors other than plasmid encapsidation by prophages. It is unknown with the data presented here whether phages actually are the selective preasure limiting plasmid size. It could be that the replication systems become unstable upon certain size, for example. More data is needed to support the claim made by the authors.

This comment aligns with a minor comment regarding Figure 4C, where transductants are presented as CFUs but no normalisation is presented, and with the sentence in Line 419.

24) Line 387. These IS mediated rearrangements have also been observed in staphylococci (doi: 10.1128/mBio.03094-20).

25) Figure 412. Based on the data presented here, it cannot be concluded whether the frequency is low or not.

26) Figure S6A. Are these transconjugants or transductants?

Reviewer #2

(Remarks to the Author)
In this study, Ipoutcha and colleagues conduct a comprehensive analysis of the evolutionary implications of multireplicon plasmids in Staphylococcus aureus, focusing on their fusion and fission dynamics. The authors first provide bioinformatic evidence demonstrating that multireplicon plasmids are abundant, enriched in insertion sequence (IS) elements, and primarily capable of conjugative transfer. They convincingly demonstrate that these plasmids form via IS-mediated recombination, yielding larger plasmids that combine multiple antibiotic resistance genes. Moreover, these plasmids can revert to their original forms or generate novel combinations spontaneously, with phage transduction selecting for these smaller variants. Collectively, their findings elucidate a life cycle that helps explain the observed genetic diversity of plasmids.

Overall, the manuscript is well-written, and the logic is generally easy to follow. The experiments and analyses are appropriate, and the conclusions drawn are sound and justified. I particularly enjoyed the phage experimental work, which provides a new angle on plasmid evolution that will be important for the field. I have some comments, mostly aimed at

improving the clarity of the manuscript:

> Figure 1b. The fact that pOriT plasmids are enriched in mutireplicon plasmids is puzzling. Why do the authors think that's the case? Also, adding statistics to this plot will help the reader assess the differences.

> Figure S1C does not differentiate plasmid size according to whether plasmids are single- or multi-replicon, so this sentence is difficult to evaluate.

> Line 129: "but RepL replicons were also present in plasmids with other replicons". I don't fully understand this sentence: RepL plasmids are by far the least involved in plasmid fusion, according to Figure S1. Please clarify.

> Line 131: Do Rep_2 plasmids show higher fusion frequencies as well? Perhaps adding full statistics to Figure S1 will help.

> I might have missed it, but I don't think Figure 2F is mentioned in the text.

> Line 169, section: "Plasmid families with conserved backbones show evidence of modular evolution". While I agree with the general interpretation of these results, I think the manuscript will benefit if the authors made clearer that they are now analyzing single-replicon plasmids.

> Line 207. I cannot find the primers in Figure S3 (except for their sequences in the supp. table), nor the rationale for their design. Provided that the plasmids carry multiple IS elements, it seems hard to predict which recombination events will be generated, and therefore, primer design seems non-trivial. An explanation of how these primers were designed, the expected band sizes, as in Figure S5 would be highly appreciated. Also, the Figure S3 legend does not clarify why there are several bands in the gel.

> I wonder whether the only homologous region between conjugative and mobilizable plasmids is the IS257 gene. Can other homologous sequences drive plasmid fusion in the experiment? This is particularly relevant in light of the statement on lines 280-282

> Line 273. What drives pNoIS fusion? As far as I understand, it bears no sequence homology nor transposases that could drive fusion.

> In Figure 4B there seem to be differences in growth rates, however the paper states that no significant differences were observed. How is this statement statistically supported? Also, it is a bit unclear where these plasmids with inactivated replication machineries come from.

> L319. Which phage was used here?

> I don't understand the difference between the experiments shown in Figure 4CD and Figure 5. My interpretation is that these two sections explore the selection of fission plasmids via transduction and investigate the outcomes of the fission products, but I don't see why they are separated into two sections in the manuscript. To me, both sections are conceptually very similar.

> Would the rate of fission be approximated from the transduction experiments, just as the authors did for the fusion rates using conjugation rates?

> In general, figure legends are too short and fail to provide sufficient context for the figures. There are also multiple typos. Please revise carefully.

Version 1:

Reviewer comments:

Reviewer #1

(Remarks to the Author)
The authors have addressed all of the points raised by the reviewer, and I appreciate the care taken in revising both the manuscript and the responses. Overall, I believe the manuscript is now suitable for publication. I only have a few minor comments for the authors to consider.

Regarding point 1, I largely agree with the authors' response. I agree that truncated rep genes and ori regions can represent signatures of historical fusion events. However, I would encourage some caution with the terminology. While these elements may indeed constitute evidence of past fusion events, they do not necessarily imply the presence of multiple active replication systems. Therefore, plasmids carrying inactive or truncated replicons may not strictly qualify as multireplicon plasmids in the functional sense. Clarifying this distinction in the manuscript could help avoid ambiguity.

Regarding point 16, the authors acknowledge that a fitness cost is observed in at least some cases. For this reason, I wonder whether the section title "Plasmid size does not impose a fitness cost after fusion" may be somewhat stronger than

warranted by the data. The results seem more consistent with the conclusion that fitness effects are context-dependent and that plasmid size alone cannot explain the observed patterns of plasmid evolution. A more nuanced wording may better reflect the data presented.

Regarding point 23, I appreciate that the authors have substantially toned down their interpretation. However, I would still encourage caution when discussing phage-mediated transfer as a “strong” selective force. For example, the statement that “phage-mediated transfer can act as a strong selective filter on plasmid size” may still overstate what is directly demonstrated. Although transduction frequencies are now presented as transductants per mL of lysate, this does not account for potential variation in the concentration of phage-derived particles between lysates. More importantly, as the authors themselves acknowledge, the association between plasmid size and phage packaging capacity remains largely correlative. The data convincingly show that phage-mediated transfer can contribute to the observed pattern, but they do not yet establish causality or quantify its relative importance compared with other factors. I therefore encourage the authors to maintain cautious wording throughout the Discussion.

Apart from these minor points, I would like to congratulate the authors on a very interesting and carefully executed study. I enjoyed reading the manuscript and believe it makes a valuable contribution to our understanding of plasmid evolution.

Reviewer #2

(Remarks to the Author)
I'm glad to see that all my comments have been successfully addressed, and I'm truly happy with the quality of the paper now. Congratulations to the authors on their wonderful work!

Open Access This Peer Review File is licensed under a Creative Commons Attribution 4.0 International License, which permits use, sharing, adaptation, distribution and reproduction in any medium or format, as long as you give appropriate credit to the original author(s) and the source, provide a link to the Creative Commons license, and indicate if changes were made.
In cases where reviewers are anonymous, credit should be given to 'Anonymous Referee' and the source.
The images or other third party material in this Peer Review File are included in the article’s Creative Commons license, unless indicated otherwise in a credit line to the material. If material is not included in the article’s Creative Commons license and your intended use is not permitted by statutory regulation or exceeds the permitted use, you will need to obtain permission directly from the copyright holder.
To view a copy of this license, visit https://creativecommons.org/licenses/by/4.0/

Response to Reviewers.

We thank both reviewers for their careful evaluation of our manuscript, for their supportive assessment of the study, and for their many constructive comments and suggestions. We greatly appreciate the time and effort invested in reviewing our work. We believe that the reviewers' feedback has significantly improved the clarity, rigor, and overall quality of the manuscript. In response, we have carefully revised the manuscript, expanded the methodological descriptions, improved the figures and figure legends, clarified several interpretations, moderated statements that could be overinterpreted, and incorporated additional analyses and discussion points as suggested. Below, we provide a detailed point-by-point response to all comments. Reviewer comments are shown in black and our responses are provided in blue.

Reviewer #1.

The authors present a study on plasmid evolution in *Staphylococcus aureus*, proposing that mobile genetic elements (MGEs) drive a cyclical fusion–deletion process that shapes plasmid diversity. The work combines large-scale genomic analyses with experimental work to support a model in which MGE-mediated fusion events generate plasmids with expanded gene content, while opposing selective pressures favour deletion derivatives. Overall, the study addresses an important question in plasmid evolution and provides a compelling conceptual framework.

My area of expertise is bacterial genetics, and therefore my review focuses primarily on this aspect.

We thank reviewer 1 for the introduction and for reviewing this paper. We greatly appreciate the comments, which help improve the quality of the manuscript.

1) Line 119. In the analysis used to define a plasmid as multireplicon, did you consider not only the presence of multiple rep genes but also their associated origins of replication (ori)?

Replicon types were identified using PlasmidFinder, which detects both replication genes and their associated oriV sequences when available. Each replicon type in the database is represented by a curated entry that includes the rep gene together with the corresponding oriV region.

PlasmidFinder is widely used and actively curated, representing one of the most comprehensive plasmid replicon databases currently available. However, not all oriV regions have been experimentally characterised, and therefore some database entries lack complete oriV information. While this represents a limitation of the field, it does not affect the main conclusions of our study. In our analyses, we only considered replicons identified with high confidence (identity >80% and coverage >60%). Moreover, because truncated or inactive *rep* genes can still represent signatures of historical fusion events, the presence of multiple fully functional oriV systems was not required for classification as multireplicon plasmids.

2) Line 187. The text refers to Figure 2C, but if I am not mistaken, it should refer to Figure 2B.

Corrected.

3) Line 188. The 6% of plasmids larger than 45 kb, are they using the same ori as the smaller plasmids, or do they represent a distinct group?

The same *ori* can be find on small and large plasmid, large plasmids represent various types of plasmids. Among the 162 plasmids, Rep1, Rep3, Rep_trans, RepA_N, and Inc18 families are present, with 24 Rep subfamilies and various multireplicon combinations. No mandatory Rep family/subtype is observed.

We have now added a figure in supplementary material with the size distribution of the replicons Fig S1C and S1D and change Fig. 2B and 2F.

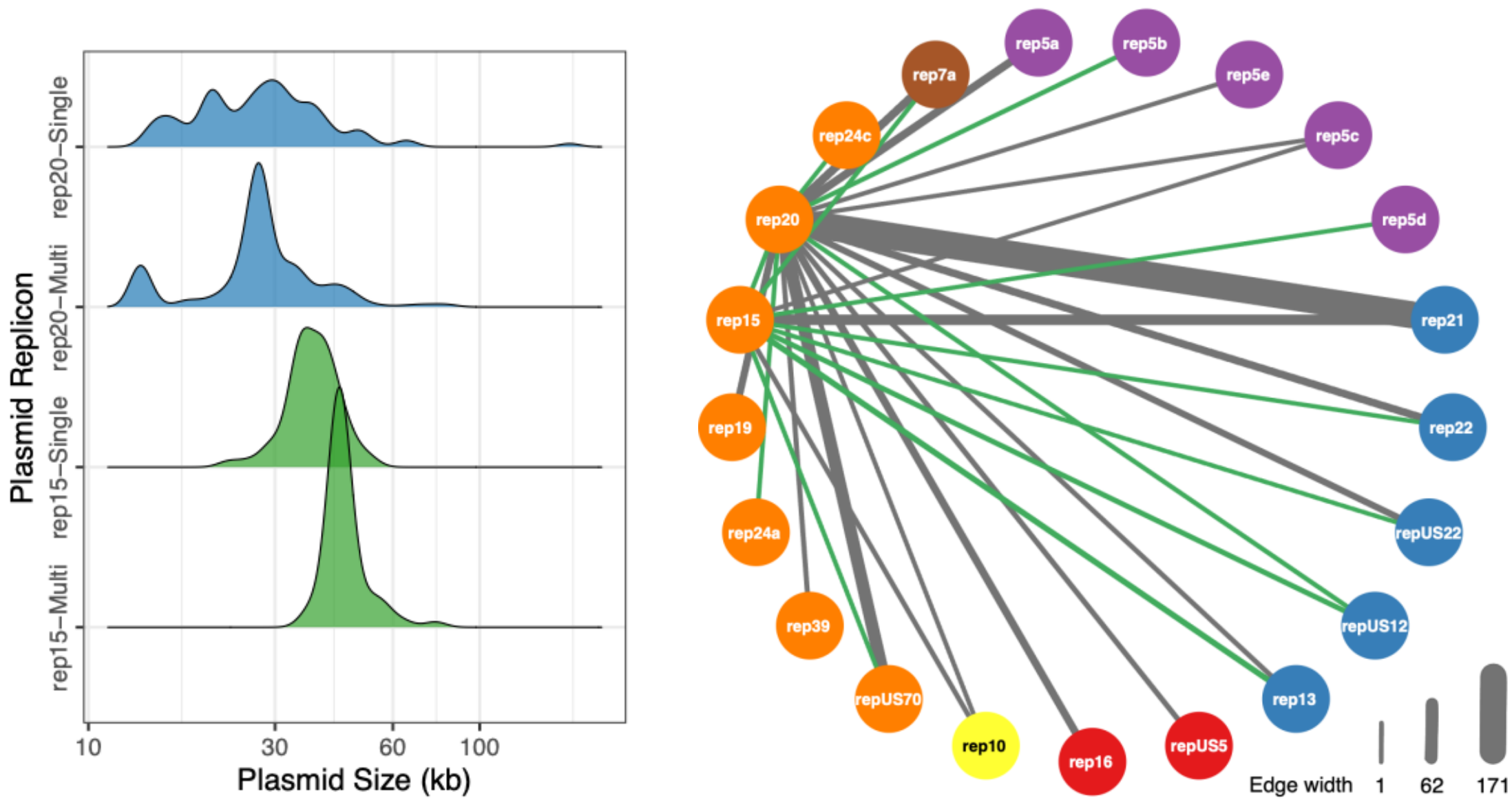

Plasmid replicon combinations (edges) are colored in green for median size > 45 kbp and grey for median size ≤ 45 kbp.

4) Lines 230–231. The Materials and Methods section does not describe how the conjugation experiments were performed (solid or liquid mating, incubation time, donor:recipient ratio, etc.). In addition, the way transconjugants are presented (Figures 3B, 5A, S3B, S4B) is not standard in the literature. Several calculation methods can be used (some accounting for donor numbers and mating time), but the simplest is to express results as the number of transconjugants divided by the total number of recipients. As currently presented, the data do not provide clear information about transfer frequencies.

We apologise for the lack of methodological detail. A complete description of the conjugation assays, including mating conditions, incubation times, and donor-to-recipient ratios, has now been added to the Methods section. In addition, to follow standard practice in the field, conjugation efficiencies are now presented as the number of transconjugants relative to the total number of recipients. All corresponding figures and legends have been revised accordingly.

5) Regarding the Materials and Methods, some plasmids were constructed by yeast-mediated assembly, but it is unclear how the YAC backbone was removed from the final plasmid. Please clarify.

For the construction in yeast, the YAC backbone is composed of an autonomously replicating sequence (ARS), a centromeric sequence (Cen6), and an auxotrophic marker (TRP1), representing 1.7 kb of extra DNA that is retained in the final version of the plasmid in *Staphylococcus aureus*.

To clarify the strategy, we added to the Methods section the following sentence L538-539: "1.7 kb DNA scars remain on the plasmid and include YAC elements for yeast replication and selection."

6) Figure S3C should be moved to the main figures and presented together with Figure 3B.

We performed appropriate changes as suggested.

7) Figures 3D and S3D should be combined into a single figure. Figure S3D also needs to be better organised, and the legend should be clarified.

We made the appropriate changes to improve figure clarity and address comments 6 and 7. Additionally, all figure legends have been revised and improved.

8) Figure legends in general need improvement. For example, avoid false friends such as “experience,” and provide sufficient detail so that the reader does not need to infer key information. The legends should be more self-explanatory.

Following the reviewer’s comment, all figure legends have now been revised and improved.

9) Lines 243–245. This is an extremely interesting observation that is not discussed at all by the authors. If rep13 (a high-copy-number replicon, as described by the authors) is not mutated, this suggests some level of regulation between replication systems in fused plasmids. One might expect the copy number of a fused plasmid to reflect that of the highest-copy replicon present; however, this is not observed (figure S6). Instead, plasmids containing a high-copy-number rep display a low copy number overall. This strongly suggests regulatory interactions between replicons, possibly involving dominance or recessiveness effects. This point is highly interesting and deserves discussion and, in my opinion, future research.

We fully agree with the reviewer that this is a particularly interesting observation. Fusion plasmids consistently tended to adopt the copy number of the lower-copy-number replicon, suggesting regulatory interactions between replication systems within multireplicon plasmids. A similar phenomenon was previously noted in the computational study by Ramiro-Martínez et al. (Figure 4), although involving different replicon combinations.

While experimentally dissecting these regulatory interactions would be highly valuable, we believe this question falls outside the scope of the present study. To acknowledge this point more clearly, the manuscript has been revised as follows:

L248: “suggesting regulatory interactions between replication systems that favour the dominance of lower-copy-number replicons in multireplicon plasmids”

In addition, the following sentence has been added to the Discussion (L469):

“Additionally, the reduced copy number observed in multireplicon plasmids points to the existence of intriguing inter replicon regulatory system that results in plasmid copy numbers lower than those of the highest copy-number plasmid. This warrants further investigation since it may shape the fate of fusion events and modulate plasmid incompatibility in multireplicon backgrounds.”

10) Line 266. Why is Figure S4C introduced before Figures S4A and S4B?

Corrected.

11) Line 273. Introduce the name of plasmid pHR.

p.HR is now introduced in the text at L275: “as well as a variant containing an alternative region of homology (p.HR, containing the trimethoprim resistance gene shared with p.EvoF and p.731)”.

12) Line 273. Selective conditions do not favour fusion per se; rather, they select for fusion events that have already occurred.

We thank Reviewer 1 for pointing this out. Indeed, our data do not support this interpretation, and we additionally show through PCR that fusion events occur naturally in the population. We therefore corrected the sentence in line 278, and “under selective conditions favouring fusion” has now been replaced with “under antibiotic pressure.”

13) Line 278. This should refer to Figure S4E, not S4C–S4D.

Corrected.

14) Line 280. Define what is meant by “short.”

The text has been changed and specifies L289 “(>600 bp)”, which corresponds to the size of an IS*257*, allowing us to observe fusion events by homologous recombination.

15) Line 298. According to the figure, the plasmid is 79 kb. Otherwise, the 76 kb plasmid corresponds to the pEVO derivative.

We thank Reviewer 1 for pointing this, text has been changed and refer to p.EvoF and not pGO1 anymore:

L307: "The 76-kb fused plasmid derived from p.EvoF grew comparably to its parental plasmids pGO1 (54 kb) and p.EvoF (42 kb)".

16) Line 299. The blue line (pGO1 + p144) indicates a fitness cost, with reduced total growth and a longer lag phase.

In general, for most selected plasmids, we do not observe a significant fitness cost between fused, original, and reduced plasmids (Fig. 4, 5, and S6). Indeed, several cases show only a slight reduction, or even the opposite effect, as observed for p.731, where the original strain grows more slowly. Overall, this supports the interpretation that fitness is not the main driver of deletion selection in our system.

To clarify that we considered and interpreted all results, the following sentence has been added.

L310-311: "Only the 79-kb fused plasmid derived from pGO1 indicates a small fitness cost, with reduced total growth and a longer lag phase."

17) Line 302. The plasmid is 77 kb according to the figure.

Corrected.

18) Line 308. There are differences in pGO1: the version carrying two rep genes (red line) shows reduced total growth.

In one case, the presence of two active rep genes is slightly deleterious, whereas in another case the effect is reversed. Overall, this supports the interpretation that the presence of multiple functional rep genes is not the main driver of deletion selection in our system. As previously noted, this also suggests a complex and interesting interaction between replicons that warrants further investigation in future work.

To ensure that all results are properly interpreted and clearly presented, the following sentence has been added (L317–319): "No consistent growth differences were observed: in one case, pGO1 carrying two active rep genes showed a slight growth reduction, whereas in another, p.731 with two active rep genes displayed a modest fitness advantage."

19) Line 323. Remove the reference to Figure 4C.

The reference has been removed.

20) Line 342. This point has already been demonstrated in the previous section.

We apologise for the lack of clarity. Although the experimental approaches are related, the objectives of the two experiments are distinct.

Figure 4C focuses on plasmid resolution following a single round of phage infection. In this experiment, selection was applied for the erythromycin marker carried by p.3912, with the expectation that only resolved forms of the original small plasmid would be recovered, since the fused plasmids exceed phage packaging capacity. However, several recovered colonies retained additional resistance markers from the conjugative plasmid, indicating the formation of novel deletion derivatives rather than simple plasmid resolution.

In contrast, Figure 5 was specifically designed to select for newly generated intermediate-size plasmid derivatives. This larger experiment included multiple fusion combinations, two different phages, and modified selection conditions targeting either the conjugative plasmid marker alone or both plasmid markers simultaneously. Because neither the conjugative plasmids nor the fused plasmids can be directly packaged, the recovered colonies necessarily contained newly generated plasmid derivatives.

We believe these experiments address complementary aspects of plasmid diversification and therefore are best presented separately. To improve clarity, the differences between the experimental designs are now explained more explicitly in the main text and figure legends.

21) Line 371. Remove "transiently." The experiments performed do not constitute evolutionary assays, and therefore the long-term fate of newly generated plasmids is unknown.

The text has been changed as suggested. The original idea was that, since large plasmids are rarely observed in nature, this suggests that large plasmid forms may be transient intermediates before diversification. However, this interpretation may indeed be too speculative.

22) Line 373. "These fused plasmids do not represent…" This statement should either be made more speculative or supported with additional evidence. Fused plasmids can be found in databases; for example, pWBG731 carries two functional rep genes. Without direct evolutionary analysis or population studies, it is not possible to conclude that these fused plasmids do not persist.

As explained in the previous comment, our statement is based on the observation that only 6% of plasmids are larger than 45 kb, whereas approximately 50% display evidence of fusion events. However, we agree that this alone may not be sufficient to support such a strong conclusion, and the sentence has therefore been revised as follows L388-391:

"While multireplicon plasmids resulting from plasmid fusion are common in nature (~50%), only 6% of plasmids are larger than 45 kb in Staphylococcus. This suggests that plasmid fusions are accompanied either by fission or gene deletion events (or both) that drive plasmid diversification."

23) Line 376. The authors place strong emphasis on phage-mediated transduction, but quantitative data are needed to support this claim. Are there active mechanisms promoting phage encapsidation of these plasmids (e.g., pac or pac-like sites), or does transfer occur via generalized transduction? What proportion of capsids carry plasmid DNA?

Capsids may contain: phage genomes, SaPIs (if present in the strain), DNA derived from lateral transduction, chromosomal DNA via generalized transduction or plasmid DNA. So, unless otherwise proven, the observed plasmid sizes could reflect additional factors other than plasmid encapsidation by prophages. It is unknown with the data presented here whether phages actually are the selective preasure limiting plasmid size. It could be that the replication systems become unstable upon certain size, for example. More data is needed to support the claim made by the authors.

This comment aligns with a minor comment regarding Figure 4C, where transductants are presented as CFUs but no normalisation is presented, and with the sentence in Line 419.

We thank the reviewer for this important comment and agree that our data do not formally demonstrate that phage predation is the sole or universal force constraining plasmid size in nature. Our intention was instead to show that, in the experimental system studied here, phage-mediated transduction provides a strong and reproducible selective pressure favouring intermediate-size plasmids. We have therefore revised the manuscript to better acknowledge that additional factors, including plasmid instability, copy-number effects, and fitness costs, may also contribute to plasmid size limitation in different systems.

To clarify the experimental interpretation:

- All transduction data are now normalised and presented as transductants per mL of lysate.
- Plasmid transfer by phages is generally considered a form of generalized transduction mediated through recognition of pseudo-pac sequences by the phage packaging machinery. Recent work demonstrated that plasmids do not carry bona fide pac or cos sites, which likely explains their relatively low transfer frequencies

(10.1016/j.celrep.2025.116456). In the same study, addition of a pac site increased plasmid transfer efficiency by several orders of magnitude, indicating that only a small proportion of capsids naturally package plasmid DNA.

- While quantifying the precise proportion of plasmid-containing capsids would be interesting, this was not the primary objective of the present work. Rather, our goal was to determine whether phage-mediated transfer could act as a selective force favouring smaller plasmids in our experimental system.
- In our experiments, phage infection selectively enriched intermediate-size plasmid derivatives generated through homologous recombination between IS*6* elements, whereas larger fused plasmids were lost together with the lysed bacterial population.
- We also agree that additional factors may contribute to plasmid size limitation. Previous studies have shown that plasmid instability, copy-number effects, and fitness costs can all influence plasmid evolution. In our system, however, we experimentally tested several of these possibilities, including fitness costs associated with plasmid size, replicon interference, and plasmid resolution. Although modest effects were occasionally observed, only the phage-selection model consistently produced strong and reproducible results.

Importantly, our genomic analyses showed that although approximately 50% of plasmids display multireplicon architectures suggestive of fusion events, only 6% exceed 45 kb. Interestingly, this threshold closely matches the packaging capacity of the major family of transducing phages found in *S. aureus*. While correlative, this observation is consistent with the idea that phage-mediated transfer contributes to constraining plasmid size in this system.

24) Line 387. These IS mediated rearrangements have also been observed in staphylococci (doi: 10.1128/mBio.03094-20).

We have integrated this new reference into the text and modified the following sentence.

L404-406: "This role is consistent with observations in various bacterial species, where IS*6*-family elements mediate plasmid co-integration or plasmid deletion via similar mechanisms"

25) Line 412. Based on the data presented here, it cannot be concluded whether the frequency is low or not.

Presented in Figure 4C, we can observe that, in comparison to the control, we observe a 2.5 to 4.5 log difference in the number of colonies. This indicates that such deletion events (including reversion and new plasmid forms) occur in approximately one cell out of 500 to 50,000 within the population. While this is not quantitative data, this is a good indication of low-frequency events.

To avoid overinterpretation, we modified the sentence L426-430: "After fusion, plasmids can resolve into their original components or undergo deletion to generate novel chimeric plasmids retaining key functional modules."

26) Figure S6A. Are these transconjugants or transductants?

These are transductants, the figure has been corrected.

Reviewer #2.

In this study, Ipoutcha and colleagues conduct a comprehensive analysis of the evolutionary implications of multireplicon plasmids in Staphylococcus aureus, focusing on their fusion and fission dynamics. The authors first provide bioinformatic evidence demonstrating that multireplicon plasmids are abundant, enriched in insertion sequence (IS) elements, and primarily capable of conjugative transfer. They convincingly demonstrate that these plasmids form via IS-mediated recombination, yielding larger plasmids that combine multiple antibiotic resistance genes. Moreover, these plasmids can revert to their original forms or generate novel combinations spontaneously, with phage transduction selecting for these smaller variants. Collectively, their findings elucidate a life cycle that helps explain the observed genetic diversity of plasmids.

Overall, the manuscript is well-written, and the logic is generally easy to follow. The experiments and analyses are appropriate, and the conclusions drawn are sound and justified. I particularly enjoyed the phage experimental work, which provides a new angle on plasmid evolution that will be important for the field. I have some comments, mostly aimed at improving the clarity of the manuscript:

We thank reviewer 2 for the introduction and for reviewing this paper. We greatly appreciate the comments, which help improve the quality of the manuscript.

> Figure 1b. The fact that pOriT plasmids are enriched in mutireplicon plasmids is puzzling. Why do the authors think that's the case? Also, adding statistics to this plot will help the reader assess the differences.

We thank the reviewer for raising this interesting point. Statistical analyses confirmed that the distribution of single- and multireplicon plasmids across mobility classes is significantly uneven (chi-square test, $p < 0.001$), and subsequent pairwise Fisher's exact tests showed that multireplicon plasmids are specifically enriched among pOriT plasmids (odds ratio > 7, adjusted $p < 0.001$). These statistics are now included in both the main text and the figure legend.

To further explore this observation, we estimated the probability of plasmids encoding multiple replicons while controlling for plasmid size and IS content. After correction for these variables, pOriT plasmids still showed the highest probability of being multireplicon (pOriT > pMob >> pNT > pConj; all pairwise comparisons $p < 0.01$, Tukey adjusted).

One possible explanation is that deletion of the conjugation machinery provides a simple route to generate smaller plasmids that retain oriT sequences while reducing overall plasmid size. In pGO1, for example, the conjugation module is flanked by IS elements and physically separated from *oriT* (10.1128/jb.178.16.4975-4983.1996). Deletion of this large conjugation cassette (~15–22 kb) could therefore generate smaller oriT-containing multireplicon plasmids that are selectively favoured under conditions imposing size constraints, including phage-mediated transduction.

> Figure S1C does not differentiate plasmid size according to whether plasmids are single- or multi-replicon, so this sentence is difficult to evaluate.

We agree with the reviewer that the original figure did not clearly distinguish plasmid sizes according to single- versus multireplicon architecture. To address this issue without overcomplicating Figure S1C, we have now added an additional supplementary Figure S1D separating plasmid size distributions for single- and multireplicon plasmids across replicon families.

> Line 129: "but RepL replicons were also present in plasmids with other replicons". I don't fully understand this sentence: RepL plasmids are by far the least involved in plasmid fusion, according to Figure S1. Please clarify.

We apologise for the lack of clarity. Our intention was to highlight that although RepL plasmids are predominantly small single-replicon plasmids, the few larger plasmids carrying RepL almost exclusively correspond to multireplicon plasmids generated through fusion events.

To better illustrate this point, we have added Figure S1D, which complements Figure S1C. This analysis shows that single-replicon RepL plasmids are consistently very small, whereas larger plasmids carrying RepL are almost exclusively multireplicon plasmids. A similar pattern is observed for Rep_trans and Rep2 plasmids, suggesting that this trend may apply broadly across several plasmid families.

The corresponding sentence (L129–131) has now been revised as follows: "Small plasmid families, including RepL and Rep_trans, were typically restricted to 2-8 kb when present as a single replicon, whereas larger plasmids carrying these replicons were almost exclusively multireplicons that had incorporated them (Table S1, Fig. S1C, Fig. S1D)."

> Line 131: Do Rep_2 plasmids show higher fusion frequencies as well? Perhaps adding full statistics to Figure S1 will help.

Only statistically significant enrichments were highlighted in the original text. Although Rep2 plasmids showed a modest increase in fusion frequency (odds ratio = 1.2), this difference was not statistically significant ($p = 0.85$). We now state this more explicitly in the figure legend and Results section to avoid confusion.

> I might have missed it, but I don't think Figure 2F is mentioned in the text.

Thank you for noticing this omission. The text has now been revised to explicitly refer to Figure 2F: "Many accessory modules consisted of complete or partial segments from other plasmids with diverse replicon types (Fig. 2D-F). These modules were commonly flanked by IS*257* and often included additional insertion sequences, implicating transposases in both their acquisition and subsequent rearrangement."

> Line 169, section: "Plasmid families with conserved backbones show evidence of modular evolution". While I agree with the general interpretation of these results, I think the manuscript will benefit if the authors made clearer that they are now analyzing single-replicon plasmids.

We apologise for the confusion. In this section, we focused on plasmids carrying either Rep15 or Rep20 replicons in order to investigate how plasmid backbones diversify through acquisition and loss of accessory modules. Some plasmids within these families are single-replicon plasmids, whereas others are multireplicon plasmids.

The corresponding text (L175–177) and Figure 2 legend have now been revised to clarify this point more explicitly.

> Line 207. I cannot find the primers in Figure S3 (except for their sequences in the supp. table), nor the rationale for their design. Provided that the plasmids carry multiple IS elements, it seems hard to predict which recombination events will be generated, and therefore, primer design seems non-trivial. An explanation of how these primers were designed, the expected band sizes, as in Figure S5 would be highly appreciated. Also, the Figure S3 legend does not clarify why there are several bands in the gel.

We apologise for the lack of clarity. Primers were initially omitted from the figure to preserve readability, but we agree that the rationale for their design should be explained more clearly. The legends of Figures S3 and S5 have now been expanded accordingly.

Figure S3 legend now states: "Primers were designed outside the IS*6* elements, thereby providing unique binding sites on each plasmid. If fusion occurs through homologous recombination between IS*6* elements from both plasmids, amplification products of 1357 bp for pGO1–p.3912 and 1387 bp for p.731–p.3912 are expected."

Figure S5 legend now states: "Primers were designed outside the IS*6* elements, thereby providing unique binding sites flanking each IS*6* copy. If deletion occurs through homologous

recombination between IS*6* elements, amplification products of the sizes indicated in the table are expected."

In addition, the figure legends now clarify the identity of the control lanes and the origin of additional bands observed in the gels.

> I wonder whether the only homologous region between conjugative and mobilizable plasmids is the IS257 gene. Can other homologous sequences drive plasmid fusion in the experiment? This is particularly relevant in light of the statement on lines 280-282

In the experiments shown in Figure S4, we specifically designed the p.HR construct to contain a homologous region unrelated to IS*257*, corresponding instead to the trimethoprim resistance gene shared between pGO1, pWBG731, and p.HR.

Using this system, we observed that plasmid fusion could occur both through IS-mediated mechanisms (transposition) and through homologous recombination mediated by the trimethoprim resistance gene (Fig. S4). The figure legend and main text have now been revised to clarify this experimental design more explicitly.

> Line 273. What drives pNoIS fusion? As far as I understand, it bears no sequence homology nor transposases that could drive fusion.

We apologise for the lack of clarity. Although p.noIS itself does not contain IS elements or homologous regions, the conjugative plasmids pGO1 and pWBG731 still carry IS*6* elements that can mediate transposition events. In this context, IS*6*-mediated transposition from the conjugative plasmid is sufficient to promote incorporation of the smaller plasmid into the fused structure.

The text has now been revised to clarify this point: "For p.noIS, which does not contain homologous sequences or IS elements, transposition can still be mediated by the IS*6* element carried on the conjugative plasmid. In the case of p.HR, both homologous recombination and transposition were observed (Fig. S4E)."

> In Figure 4B there seem to be differences in growth rates, however the paper states that no significant differences were observed. How is this statement statistically supported? Also, it is a bit unclear where these plasmids with inactivated replication machineries come from.

We apologise for the lack of clarity. In response to comments from both reviewers, the text has now been revised to more accurately describe the observed differences between conditions.

Overall, most plasmid combinations did not display strong or consistent fitness defects associated with plasmid fusion, although modest effects were observed in some cases. For example, the 79-kb pGO1-derived fused plasmid displayed a slightly longer lag phase and reduced final growth, whereas in the p.731 background the opposite trend was observed.

Similarly, for plasmids carrying two active replication systems, one combination showed a slight fitness reduction whereas another displayed a modest fitness advantage. These observations suggest that neither plasmid size nor the presence of multiple active replication systems alone consistently explains deletion selection in our system.

In addition, the origin of plasmids carrying inactive rep genes has now been clarified in the Figure 4B legend. These plasmids originated from previous fusion experiments in which transposition events disrupted one of the replication genes.

> L319. Which phage was used here?

We apologise for the missing information. The phage used in this experiment was 80α. This information has now been added to the main text and figure legends.

> I don't understand the difference between the experiments shown in Figure 4CD and Figure 5. My interpretation is that these two sections explore the selection of fission plasmids via transduction and investigate the outcomes of the fission products, but I don't see why they are

separated into two sections in the manuscript. To me, both sections are conceptually very similar.

We apologise for the lack of clarity. Although conceptually related, the two experiments address different aspects of plasmid diversification.

Figure 4C,D focuses on plasmid resolution following a single round of phage infection. In this experiment, selection was applied for the erythromycin marker carried by p.3912, with the expectation that only resolved forms of the original small plasmid would be recovered. However, some recovered colonies retained additional resistance markers from the conjugative plasmid, indicating the formation of novel deletion derivatives rather than simple plasmid resolution.

In contrast, Figure 5 was specifically designed to select directly for newly generated intermediate-size plasmid derivatives. This larger experiment included multiple fusion combinations, two different phages, and modified selection conditions targeting either the conjugative plasmid marker alone or both plasmid markers simultaneously. Because neither the conjugative plasmids nor the fused plasmids can be directly packaged into phage capsids, the recovered colonies necessarily contained newly generated plasmid derivatives.

We believe these experiments therefore address complementary aspects of plasmid diversification and are best presented separately. To improve clarity, the differences between the experimental designs are now explained more explicitly in the main text and figure legends.

> Would the rate of fission be approximated from the transduction experiments, just as the authors did for the fusion rates using conjugation rates?

The transduction experiments provide an approximate indication that deletion and resolution events occur at low frequency within the population. In Figure 4C, we observed a 2.5–4.5 log reduction in colony numbers when the plasmid was present in the fused context compared with the small plasmid alone. Because fused plasmids cannot themselves be directly packaged into phage capsids, recovered transductants necessarily derive from deletion or resolution events that generate smaller transferable plasmids.

However, because the resulting estimates span a relatively broad range and multiple processes may contribute to the observed frequencies, we chose not to provide an explicit quantitative estimate and instead refer to these events as rare. We also note that homologous recombination itself is expected to occur at intrinsically low frequency.

> In general, figure legends are too short and fail to provide sufficient context for the figures. There are also multiple typos. Please revise carefully.

We apologise for this. Both reviewers correctly noted that the original figure legends lacked sufficient detail and contained several typographical and wording issues. All figure legends have now been substantially revised and expanded to improve clarity and provide the necessary experimental context.

Response to Reviewers.

We thank both reviewers for their careful and constructive assessment of our revised manuscript. We are pleased that they consider the manuscript suitable for publication and appreciate their positive comments about the quality and significance of the study. We have considered the remaining minor points carefully and provide our responses below.

REVIEWERS' COMMENTS

Reviewer #1 (Remarks to the Author):

The authors have addressed all of the points raised by the reviewer, and I appreciate the care taken in revising both the manuscript and the responses. Overall, I believe the manuscript is now suitable for publication. I only have a few minor comments for the authors to consider.

Regarding point 1, I largely agree with the authors' response. I agree that truncated rep genes and ori regions can represent signatures of historical fusion events. However, I would encourage some caution with the terminology. While these elements may indeed constitute evidence of past fusion events, they do not necessarily imply the presence of multiple active replication systems. Therefore, plasmids carrying inactive or truncated replicons may not strictly qualify as multireplicon plasmids in the functional sense. Clarifying this distinction in the manuscript could help avoid ambiguity.

We thank the reviewer for this helpful clarification. We agree that the detection of more than one replicon-associated sequence does not necessarily indicate the presence of multiple active replication systems. In our bioinformatic analysis, the term “multireplicon” was used operationally to describe plasmids carrying more than one identifiable replicon sequence. Some of these additional replicons may be incomplete or inactive and may therefore represent signatures of historical fusion events rather than functional replication systems. We have now clarified this distinction at the first use of the term and in the Methods. Throughout the manuscript, statements concerning multiple active replication systems are restricted to the experimentally characterised plasmids for which replicon functionality could be assessed.

Regarding point 16, the authors acknowledge that a fitness cost is observed in at least some cases. For this reason, I wonder whether the section title “Plasmid size does not impose a fitness cost after fusion” may be somewhat stronger than warranted by the data. The results seem more consistent with the conclusion that fitness effects are context-dependent and that plasmid size alone cannot explain the observed patterns of plasmid evolution. A more nuanced wording may better reflect the data presented.

We agree with the reviewer that the original section title was stronger than warranted. Although we did not detect a consistent relationship between increased plasmid size and reduced bacterial growth, a modest fitness cost was observed in one plasmid background. We have therefore revised the section title to emphasise that the fitness effects are not consistent across plasmid backgrounds. The text already acknowledges these context-dependent effects, but we have made the wording fully consistent throughout the section.

Regarding point 23, I appreciate that the authors have substantially toned down their interpretation. However, I would still encourage caution when discussing phage-mediated transfer as a “strong” selective force. For example, the statement that “phage-mediated transfer can act as a strong selective filter on plasmid size” may still overstate what is directly demonstrated. Although transduction frequencies are now presented as transductants per mL of lysate, this does not account for potential variation in the concentration of phage-derived particles between lysates. More importantly, as the authors themselves acknowledge, the association between plasmid size and phage packaging capacity remains largely correlative. The data convincingly show that phage-mediated transfer can contribute to the observed pattern, but they do not yet establish causality or quantify its relative importance compared

with other factors. I therefore encourage the authors to maintain cautious wording throughout the Discussion.

We thank the reviewer for raising this point. We agree that transductants per mL of lysate do not normalise for variation in the abundance of phage-derived particles and therefore cannot be used to compare absolute transfer efficiencies between lysates or to quantify the relative ecological importance of phage-mediated selection. However, our conclusion is not based primarily on quantitative differences in transduction frequency. It is based on the size and architecture of the plasmids recovered after transduction. Fused plasmids exceeding the phage packaging capacity were not recovered intact. Instead, across different plasmid backgrounds and two phages, transduction consistently recovered deletion or resolution derivatives of 33.5-44.5 kb, within the relevant packaging range. These derivatives showed no detectable growth advantage over their fused precursors.

Variation in particle abundance may affect the number of transductants recovered, but it cannot explain the consistent selection of plasmids compatible with phage packaging limits. We therefore believe that our experiments directly demonstrate that phage-mediated transfer can act as a strong size-dependent filter during transduction. The constrained natural plasmid size distribution, with only 6% of analysed Staphylococcus plasmids exceeding 45 kb, further supports the biological relevance of this mechanism. We agree, however, that our experiments do not quantify its relative importance compared with other evolutionary forces in natural populations.

Apart from these minor points, I would like to congratulate the authors on a very interesting and carefully executed study. I enjoyed reading the manuscript and believe it makes a valuable contribution to our understanding of plasmid evolution.

We thank the reviewer for these positive comments and for their careful and constructive assessment of our work.

Reviewer #2 (Remarks to the Author):

I'm glad to see that all my comments have been successfully addressed, and I'm truly happy with the quality of the paper now. Congratulations to the authors on their wonderful work!

We sincerely thank the reviewer for these very positive comments and for their constructive assessment of our manuscript.
