# Supplementary material for: Mobile genetic elements drive a plasmid fusion and deletion lifecycle shaping evolution and antimicrobial resistance
Source: Nat Commun. 2026 Sep 15;17:10849. doi: 10.1038/s41467-026-77678-8 (PMC13672794; doi:10.1038/s41467-026-77678-8)
Supplement: Supplementary file 2 [file 41467_2026_77678_MOESM1_ESM.pdf]

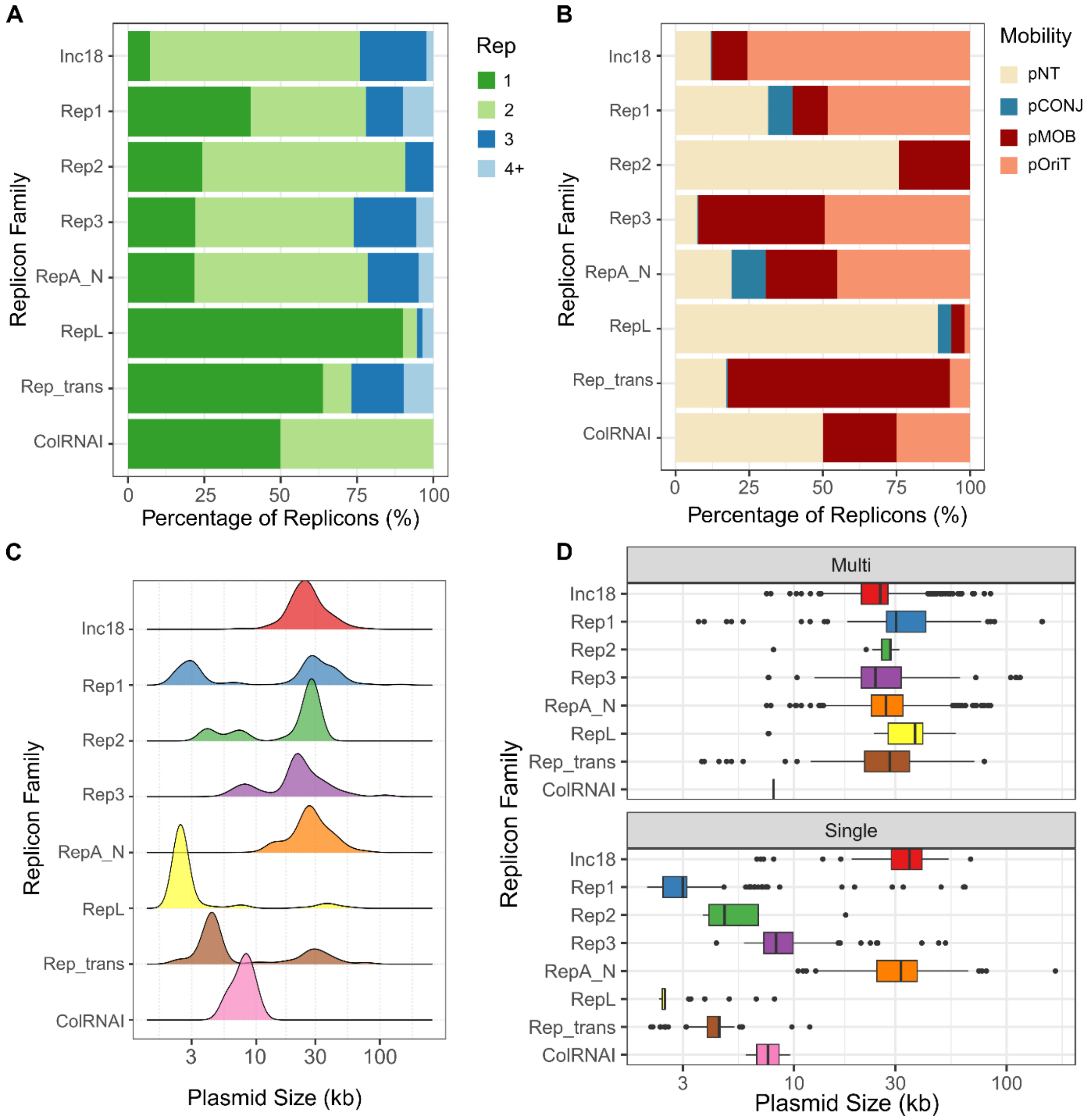


**Figure S1: Distribution of plasmid replicon families in *Staphylococcus*. A.** Proportion of plasmids encoding 1 to 4+ replicons within each replicon family. Plasmids encoding more than four replicons were grouped into the "4+" category. **B.** Distribution of plasmid mobility types across replicon families. **C.** Comparison of plasmid sizes (log10-transformed) across replicon families. **D.** Boxplots showing the size distribution of single- and multireplicon plasmids across replicon families. Multireplicon plasmids display similar size distributions across families, whereas single-replicon plasmids show greater variation between replicon families. The color scheme is the same as in Figures 1D and S1C.

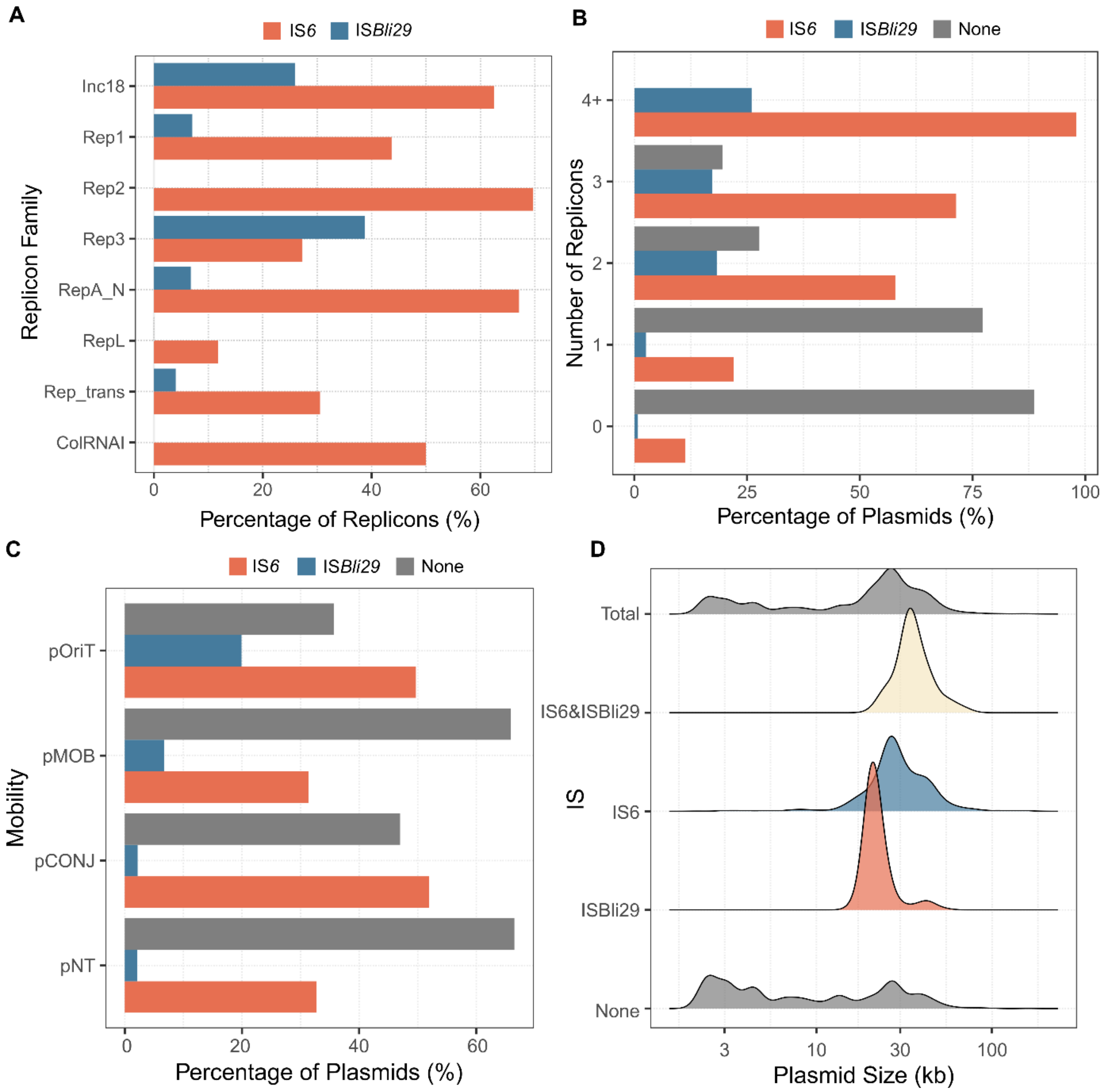


**Figure S2: Distribution of Insertion Sequence (IS) IS*6* and IS*Bli29* in *Staphylococcus* plasmids. A.** Frequency of IS*6* and IS*Bli29* in plasmids grouped by replicon family. **B.** Proportion of IS*6*- and IS*Bli29*-encoding plasmids with 1-4+ replicon(s), plasmids with more than four replicons are merged into "4+" group, plasmids encoding not identifiable replicon are in the group "0". **C.** Proportion of IS*6*- and IS*Bli29*-encoding plasmids by mobility types. **D.** Comparison of plasmid size by the presence of IS*6*, IS*Bli29*, both, and neither. (Note: 97.6% of the IS*6* here is cross-validated by ISfinder as IS*6* family clade f, i.e. IS*257*; IS*Bli29* here is the ORF4 which encodes a transposase with a Mu transposase C-terminal domain, pfam accession PF09299).

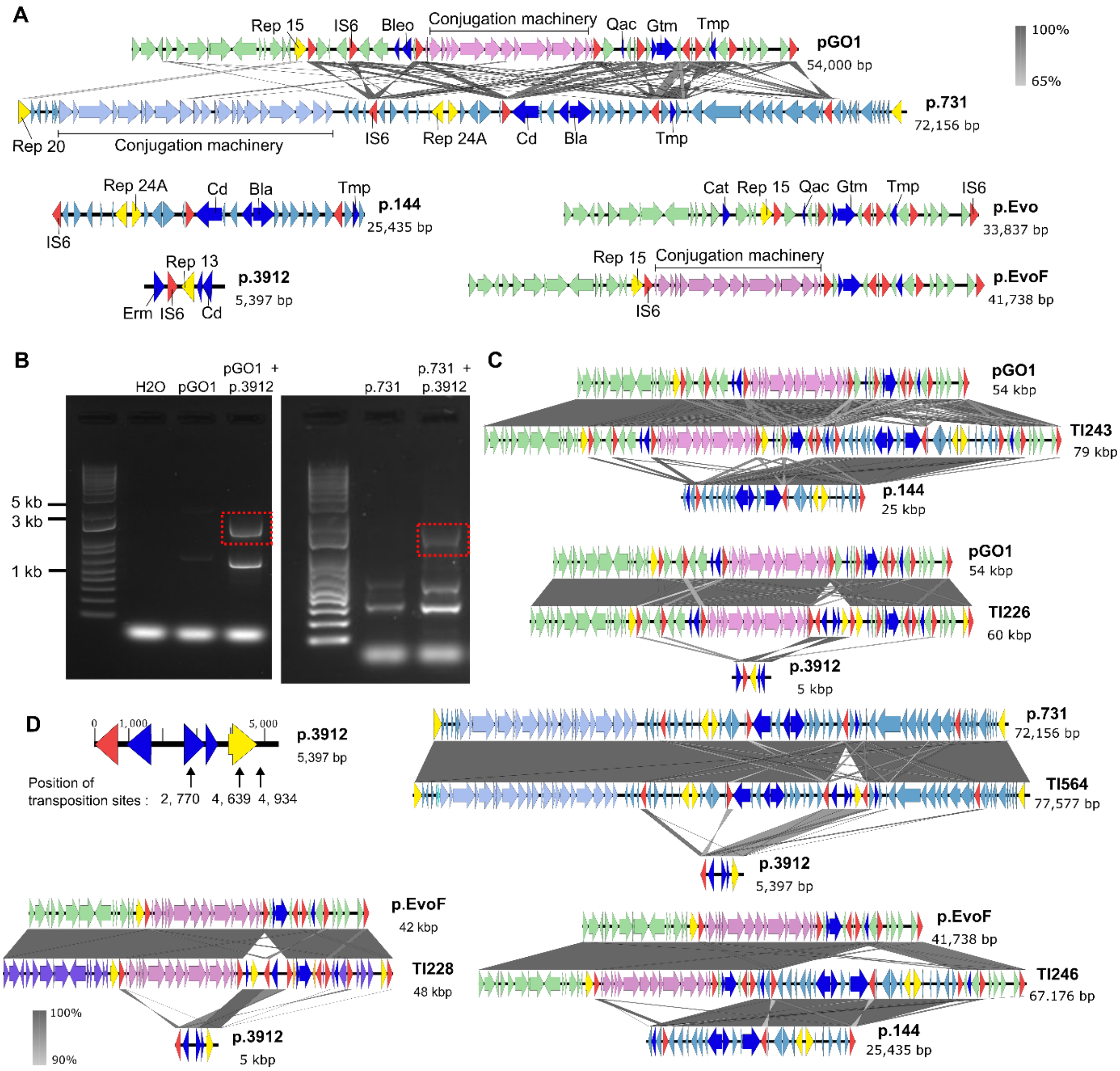


**Figure S3: Plasmid fusion assays.** *pC* (conjugative plasmid), *pNT* (non-transmissible plasmid), R (recipient). Gtm: gentamicin resistance; Cd: cadmium resistance; Tmp: trimethoprim resistance; Erm: erythromycin resistance; Bleo: bleomycin resistance; Bla: β-lactam resistance; Qac: quaternary ammonium compound resistance. **A.** Plasmid maps used in this study. **B.** Detection of fusion events between pGO1 and p.3912 or between p.731 and p.3912. Primers were designed outside the IS*6* elements, providing a unique binding site on each plasmid. If fusion occurs through homologous recombination between IS*6* elements from both plasmids, amplification products of 1357 bp for pGO1–p.3912 and 1387 bp for p.731–p.3912 are expected. Genomic DNA was extracted from cultures of RN4220 carrying a conjugative plasmid (pGO1 or p.731) with or without the small plasmid p.3912. The majority of extracted plasmids are expected to be non-fused and therefore contain only one primer-binding site. Fused plasmids are expected to be rare, consequently, a low annealing temperature (50°C) and standard Taq DNA polymerase were used to facilitate detection of these rare events. This explains the non-specific amplification products at lower size also observed without the presence of p.3912. Sanger sequencing of the PCR amplicons confirmed the recombination junctions. **C.** Additional examples of plasmid fusion events characterized by long-read sequencing. At least one plasmid from each combination was sequenced. **D.** Transposition sites identified in p.3912 from the sequenced pGO1–p.3912 and p.731–p.3912 fusion plasmids.

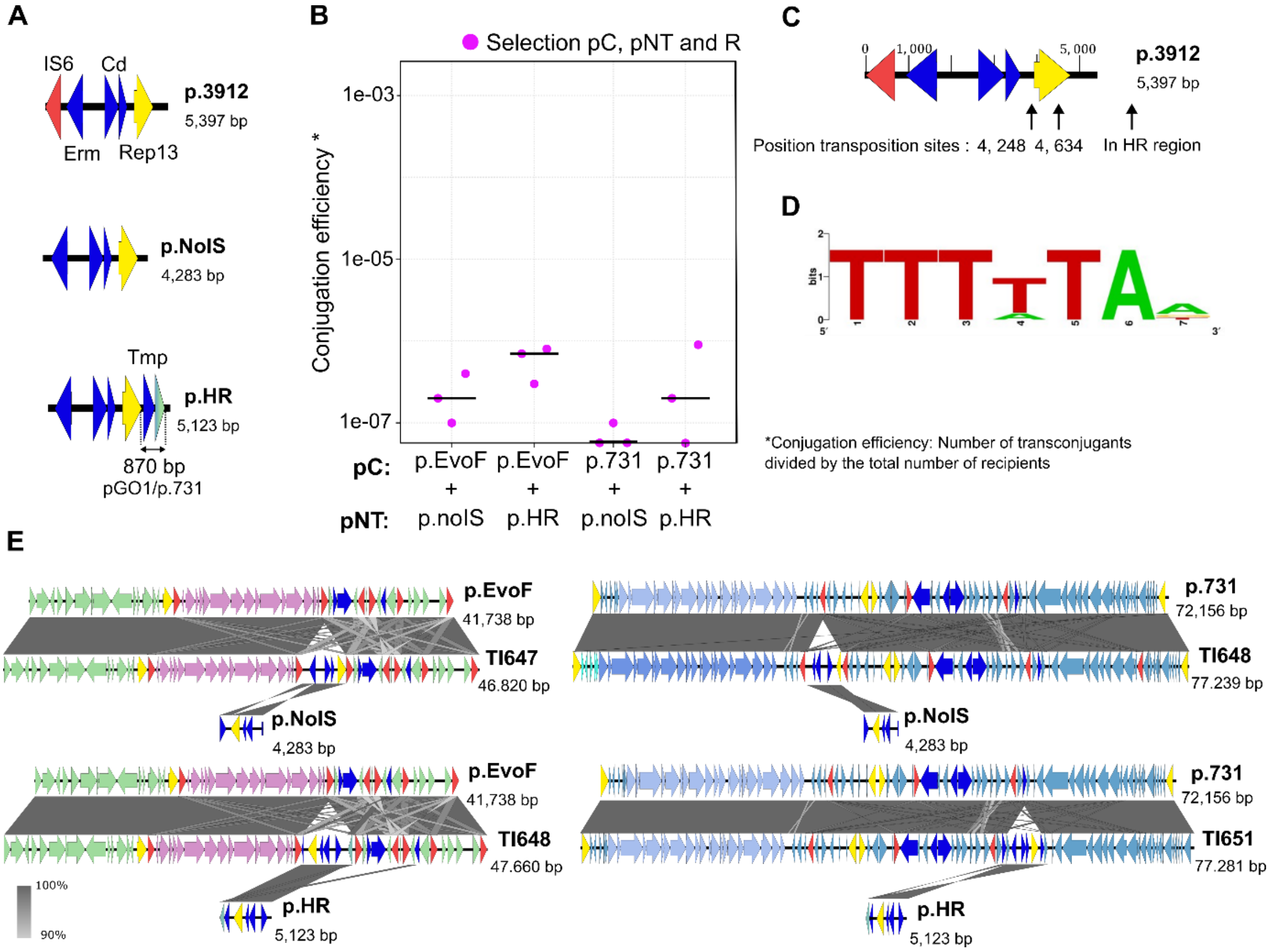


**Figure S4: Plasmid fusion is mediated from both HR or Tp events.** pC (conjugative plasmid), pNT (non-transmissible plasmid), R (recipient). Cd: Cadmium resistance, Tmp: Trimethoprim resistance and Erm: Erythromycin resistance. **A.** Derivative plasmid from p.3912 after IS*6* gene removal or replacement by another gene containing Tmp resistance and found in both pGO1 and p.731. **B.** Conjugation experiments illustrating plasmid fusion between a conjugative plasmid (p.EvoF or p.731) and p.3912 derivatives to test homologous recombination (HR) and transposition (Tp) mechanisms. The x-axis shows the plasmids present in the donor strain, and the y-axis indicates conjugation efficiency (n = 3 biological replicates; data are presented as mean values). Individual data points are shown. Selection conditions have been applied to select for pC (cadmium for p.731, gentamicin for p.EvoF), pNT (Erythromycin) and the recipient bacteria (tetracycline). **C.** Transposition sites identified in p.NolS and p.HR from the sequenced fusion plasmids. **D.** Consensus motif for transposition sites observed in p.3912 and derivatives p.nolS and p.HR, for the different sequenced clones. **E.** Additional examples of plasmid fusion events characterized by long-read sequencing. At least one plasmid from each combination was sequenced.

A

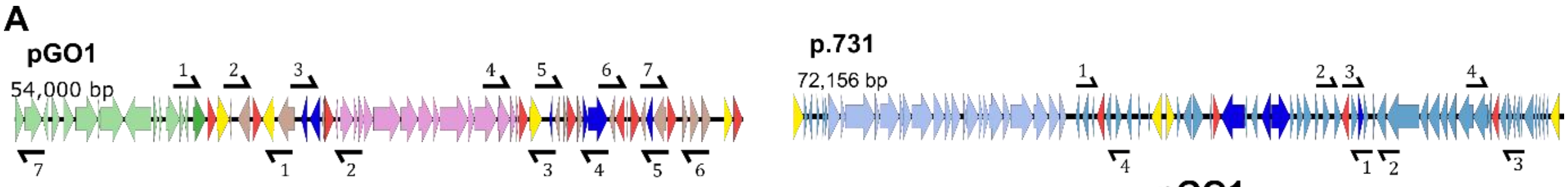


C

| | Primers | WT size | Rec.size | Amp | Sanger val. |
|---|---|---|---|---|---|
| pGO1 | 1 | 4284 | 932 | Y | nd |
| | 2 | 6345 | 999 | Y | nd |
| | 3 | 15377 | 974 | Y | nd |
| | 4 | 4948 | 1384 | Y | Y |
| | 5 | 5682 | 988 | Y | Y |
| | 6 | 4081 | 1346 | Y | Y |
| | 7 | 5925 | 1034 | Y | nd |
| | 1F/2R | 9699 | 1001 | Y | nd |
| | 1F/4R | 28075 | 1410 | Y | nd |
| | 1F/7R | 40042 | 1057 | N | nd |
| p.731 | 1 | 23822 | 924 | Y | Y |
| | 2 | 3195 | 626 | Y | Y |
| | 3 | 12617 | 1079 | Y | Y |
| | 4 | 36347 | 1148 | Y | nd |
| | 1F/2R | 26108 | 667 | Y | nd |
| | 2F/4R | 50357 | 1097 | Y | nd |
| | 3F/4R | 47928 | 1211 | Y | nd |

B

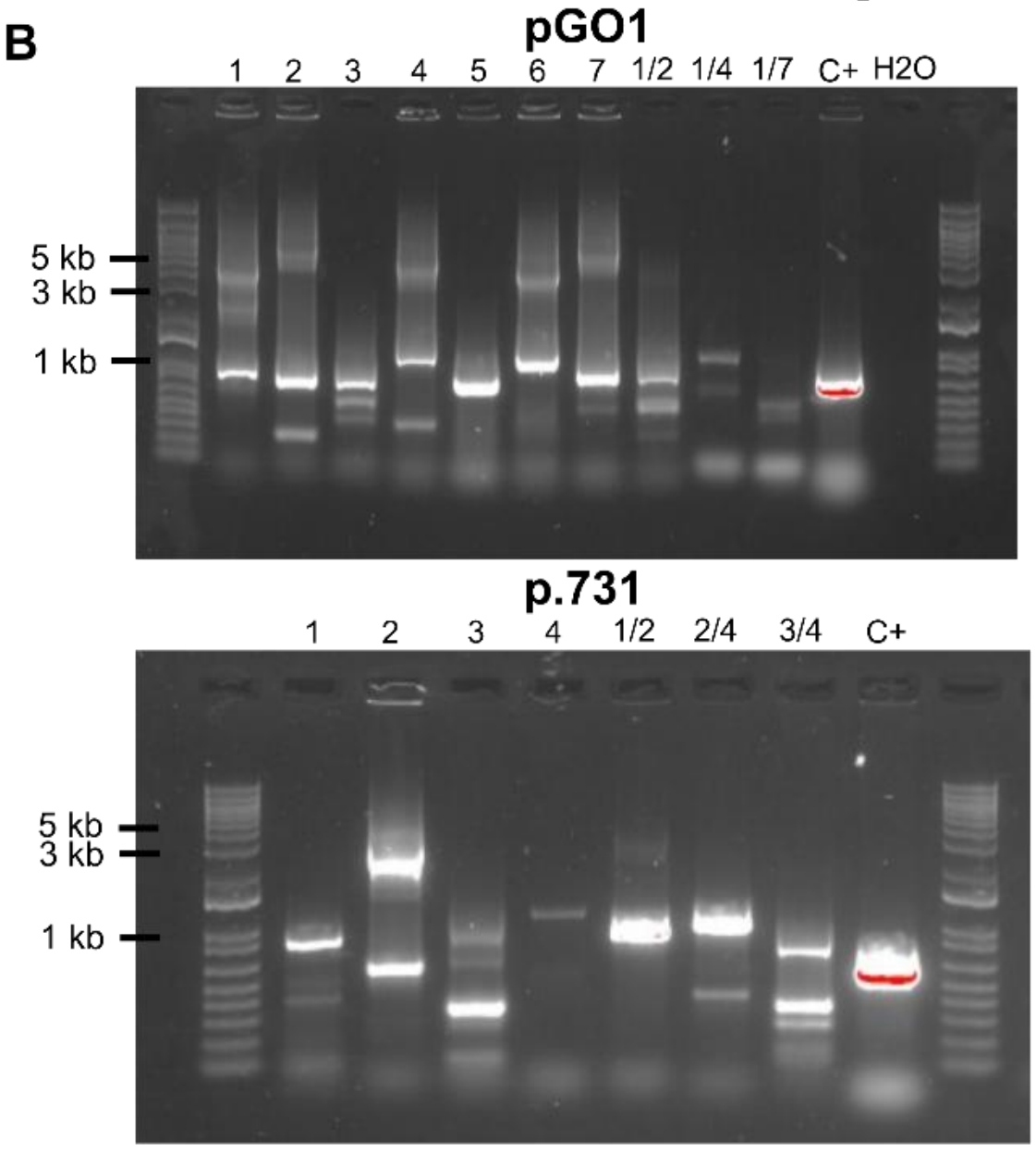


**Figure S5: Detection of deletion events in bacterial cell population. A.** Design of primers used for the detection of deletion events in pGO1 and p.731 between different IS6 copies. Primers were designed outside the IS6 elements, providing unique binding sites flanking each IS6 copy. If deletion occurs through homologous recombination between IS6 elements, amplification products of the sizes indicated in the table are expected. **B,C.** Detection of internal deletion events in pGO1 and p.731. Genomic DNA was extracted from cultures of RN4220 carrying pGO1 or p.731. The majority of extracted plasmids are expected to correspond to the WT plasmid and therefore produce high-molecular-weight DNA products, as sometimes observed on the gel. Deletion events are expected to be rare and consequently, a low annealing temperature (50°C) and standard Taq DNA polymerase were used to facilitate detection of these rare events. This likely explains the non-specific amplification products observed at lower size for several conditions. Sanger sequencing of the PCR amplicons confirmed the recombination junctions for the indicated conditions. C+ corresponds to the positive control amplification for pGO1_D_1F and pGO1_D_4R directly on p.EvoF plasmid.

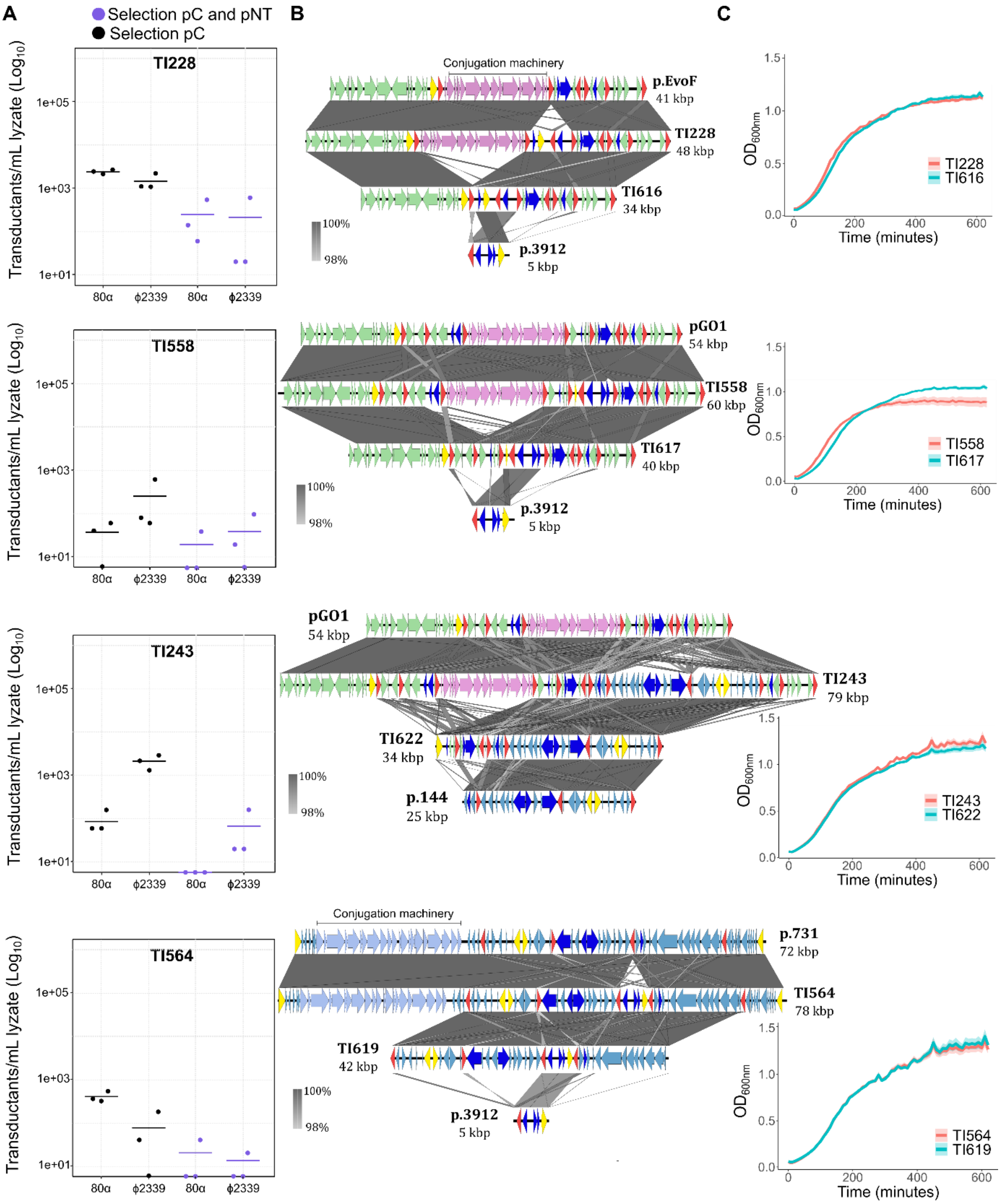

A
Selection pC and pNT
Selection pC
B
C
TI228
TI558
TI243
TI564
Transductants/mL lyzate (Log10)
1e+05
1e+03
1e+01
80α
ϕ2339
Conjugation machinery
p.EvoF
41 kbp
TI228
48 kbp
TI616
34 kbp
p.3912
5 kbp
pGO1
54 kbp
TI558
60 kbp
TI617
40 kbp
TI243
79 kbp
TI622
34 kbp
p.144
25 kbp
p.731
72 kbp
TI564
78 kbp
TI619
42 kbp
100%
98%
OD600nm
Time (minutes)
TI616
TI617
TI622
TI619

**Figure S6: Phage selects for diverse intermediate-size plasmid derivatives. A.** Selection assay for plasmid derivatives. Transduction efficiency by phage 80α or Φ2339 of the different combination of fused plasmids. Transduction of: TI228, the fused version p.EvoF/p.3912, TI558 the fused version pGO1/p.3912, TI243 the fused version pGO1/p.144 and TI564 the fused version p.731/p.3912. Black dots represent selection for only the large conjugative plasmid marker, whereas purple dots represent selection for both the conjugative and non-transmissible plasmid markers. The x-axis shows the phage used to infect the donor RN4220 strain carrying the fused plasmid. The y-axis indicates transduction efficiency ($n$ = 3 biological replicates; data are presented as mean values). Individual data points are shown. Conjugative and fused plasmids are too large to be packaged by phage 80α and Φ2339 (headful packaging limit: 41 ± 2.5 kb); therefore, selection with one or two antibiotics leads to the recovery of novel plasmid derivatives rather than the original resolved plasmids. **B.** Sequence alignment of plasmid derivatives obtained after transduction. **C.** Growth of RN4220 strains harboring the fused plasmid or its smaller derivative selected after transduction. Growth was monitored by measuring optical density at 600 nm (OD600) over 12 h of incubation ($n$ = 3).
