# Supplementary material for: Mobile genetic elements drive a plasmid fusion and deletion lifecycle shaping evolution and antimicrobial resistance
Source: Nat Commun. 2026 Sep 15;17:10849. doi: 10.1038/s41467-026-77678-8 (PMC13672794; doi:10.1038/s41467-026-77678-8)
Supplement: Supplementary file 3 [file 41467_2026_77678_MOESM2_ESM.pdf]

Description of Additional Supplementary Files

File Name: Supplementary Data 1
Description: Staphylococcus plasmids analysis.

File Name: Supplementary Data 2
Description: Staphylococcus plasmids replicon classification.

File Name: Supplementary Data 3
Description: Staphylococcus plasmids IS detection.

File Name: Supplementary Data 4
Description: Staphylococcus plasmids IS257 detections.

File Name: Supplementary Data 5
Description: Strains.

File Name: Supplementary Data 6
Description: Copy number.
